# Functionalization of Betulinic Acid with Polyphenolic Fragments for the Development of New Amphiphilic Antioxidants

**DOI:** 10.3390/antiox10020148

**Published:** 2021-01-20

**Authors:** Joana L. C. Sousa, Cristiana Gonçalves, Ricardo M. Ferreira, Susana M. Cardoso, Carmen S. R. Freire, Armando J. D. Silvestre, Artur M. S. Silva

**Affiliations:** 1 LAQV-REQUIMTE, Department of Chemistry, University of Aveiro, 3810-193 Aveiro, Portugal; csng@ua.pt (C.G.); ric.ferreira@ua.pt (R.M.F.); susanacardoso@ua.pt (S.M.C.); 2 Department of Chemistry, CICECO–Aveiro Institute of Materials, University of Aveiro, 3810-193 Aveiro, Portugal; cfreire@ua.pt (C.S.R.F.); armsil@ua.pt (A.J.D.S.)

**Keywords:** betulinic acid, oleanane-type compounds, polyhydroxylated compounds, amphiphilic antioxidants, radical scavenging activity, antioxidant activity

## Abstract

The present work aimed at the valorization of biomass derived compounds by their transformation into new added-value compounds with enhanced antioxidant properties. In this context, betulinic acid (BA) was decorated with polyphenolic fragments, and polyhydroxylated (*E*)-2-benzylidene-19,28-epoxyoleanane-3,28-diones **4a**–**d** were obtained. For that, the synthetic strategy relied on base-promoted aldol condensation reactions of methyl betulonate, which was previously prepared from natural BA, with appropriate benzaldehydes, followed by cleavage of the methyl protecting groups with BBr_3_. It is noteworthy that the HBr release during the work-up of the cleavage reactions led to the rearrangement of the lupane-type skeleton of the expected betulonic acid derivatives into oleanane-type compounds **4a**–**d**. The synthesized compounds **4a**–**d** were designed to have specific substitution patterns at C-2 of the triterpene scaffold, allowing the establishment of a structure-activity relationship. The radical scavenging ability of **4a**–**d** was evaluated using the 2,2-diphenyl-1-picrylhydrazyl radical (DPPH^•^) and 2,2′-azino-bis(3-ethylbenzothiazoline-6-sulfonic acid radical cation (ABTS^•+^) scavenging assays. In particular, derivative **4c**, bearing a catechol unit, revealed to be the most efficient scavenger against both free radicals DPPH^•^ and ABTS^•+^. Subsequently, we designed two analogues of the hit derivative **4c** in order to achieve more potent antioxidant agents: (i) the first analogue carries an additional unsaturation in its lateral chain at C-2 (analogue **5**) and (ii) in the second analogue, E-ring was kept in its open form (analogue **6**). It was observed that the presence of an extended π-conjugated system at C-2 contributed to an increased scavenging effect, since analogue **5** was more active than **6**, α-tocopherol, and **4c** in the ABTS^•+^ assay.

## 1. Introduction

**Figure 1:**
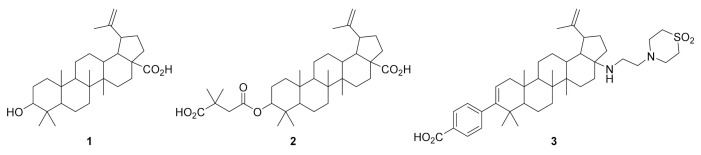
Chemical structures of BA (**1**) and the relevant synthetic BA derivatives bevirimat (**2**) and BMS-955176 (**3**).

Betulinic acid (BA, **1**) is a lupane-type pentacyclic triterpenoid (Figure 1). It can be isolated from several sources, but the most reported one is the bark of birch trees (*Betula* spp.). This triterpenic acid displays important biological properties such as anticancer, antiviral, antibacterial, and antimalarial activities, among others [1,2]. Furthermore, BA (**1**) has become one of the most studied triterpenes, since some of its synthetic derivatives, namely bevirimat (**2**) and BMS-955176 (**3**) (Figure 1), proved to be efficient anti-HIV drugs, reaching phase IIb clinical trials [3,4].

All these evidences encouraged medicinal and organic chemists to start looking to BA (**1**) as an interesting template for further chemical improvement in order to design new biologically active molecules for different applications [5]. Among them, the anticancer activity should be highlighted due to the high number of recent papers and patents referring the potential of synthetic derivatives of BA (**1**) as antitumor agents [6,7,8]. In particular, in 2017, Gupta et al. found that some synthetic analogues of BA (**1**) and betulonic acid, bearing benzylidene-type substituents at C-2, revealed a promising cytotoxic profile against various cancer cell lines (IC_50_ 1–2 µM) [9].

Free radicals as well as reactive oxygen (ROS) and nitrogen (RNS) species play an important role in the development of cancer and other diseases [10,11]. Therefore, the search for new antioxidant agents is still a current topic of intense research. Antioxidants can be divided according to their solubility. Liposoluble antioxidants, mainly vitamins A and E, have demonstrated a therapeutic and preventive role in skin care, neurodegenerative diseases, as well as cardiovascular disease and aging [12,13,14]. On the other hand, flavonoids are well-known water-soluble antioxidants [15,16]. Their antiradical activity relies especially on some of their structural features: the 3,4-dihydroxy substitution (catechol unit) in the B-ring, the C2=C3 double bond conjugated with the carbonyl group at C-4, and free hydroxy groups at C-3 and C-5 [15,16,17]. In addition, 2-styrylchromones are another interesting family of synthetic antioxidants, and comparing their scavenging effect with the correspondent flavones, the contribution of the styryl moiety to the molecular stabilization increases the compound’s antiradical activity [18,19,20].

**Figure 2:**
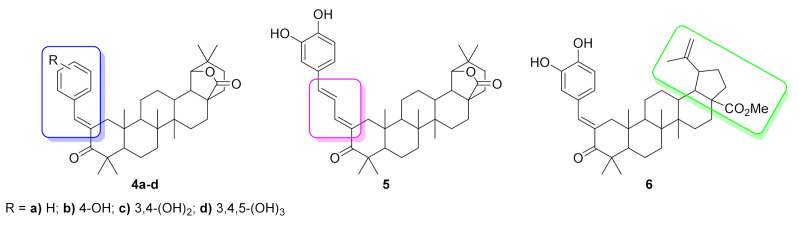
Chemical structures of the target compounds.

Bringing all this information together and aiming at the valorization of biomass derived compounds, herein, the functionalization of BA (**1**) with phenolic units was conducted to obtain amphiphilic antioxidants. Thus, polyhydroxylated (*E*)-2-benzylidene-19,28-epoxyoleanane-3,28-diones **4a**–**d** and two **4c** analogues—(*E*,*E*)-2-[3-(3,4-dihydroxyphenyl)allylidene]-19,28-epoxyoleanane-3,28-dione (**5**) and methyl (*E*)-2-(3,4-dihydroxybenzylidene)betulonate (**6**) (Figure 2)—were synthesized. These compounds were designed to present different structural features such as (i) variations in the number and position of the hydroxy groups (compounds **4a**–**d**); (ii) the presence of catechol moieties (compounds **4c**, **5**, and **6**); (iii) an extended π-conjugated system at C-2 (compound **5**); and (iv) the E-ring in its open form similar to the pristine BA (**1**) (compound **6**). Then, all these compounds were submitted to a preliminary scavenging activity evaluation against the 2,2-diphenyl-1-picrylhydrazyl radical (DPPH^•^) and 2,2′-azino-bis(3-ethylbenzothiazoline-6-sulphonic acid) radical cation (ABTS^•+^).

## 2. Materials and Methods

### 2.1. Chemistry

#### 2.1.1. General Remarks

Melting points were measured with a Büchi Melting Point B-540 apparatus and are uncorrected. NMR spectra were recorded with Bruker Avance 300 (300.13 MHz for ^1^H and 75.47 MHz for ^13^C) or Bruker Avance 500 (500.13 MHz for ^1^H and 125.77 MHz for ^13^C) spectrometers, in CDCl_3_, DMSO-*d*_6_, and acetone-*d*_6_ as solvents. Chemical shifts (δ) are reported in ppm and coupling constants (*J*) in Hz; the internal standard was tetramethylsilane (TMS). Unequivocal ^13^C assignments were made with the aid of 2D *g*HSQC and *g*HMBC (delays for one-bond and long-range *J* C/H couplings were optimized for 145 and 7 Hz, respectively) experiments. Positive ESI mass spectra were acquired with an LTQ Orbitrap XL spectrometer. Preparative thin layer chromatography (TLC) was performed with Macherey–Nagel silica gel G/UV254. Column chromatography was performed with Acros Organics silica gel 60 Å (0.060–0.200 mm). All chemicals and solvents were obtained from commercial sources and used as received or dried by standard procedures. BA (**1**) was purchased from Aktin Chemicals, Inc. (China). For intermediates **9b**–**d**, **12**, and **14** (Figure S1), ^1^H and ^13^C NMR assignments are only presented for the substituent “R”, since these spectra were all recorded in CDCl_3_ and the methyl betulonate scaffold (C1–C30) is similar to intermediate **9a**, where it was completely assigned.

#### 2.1.2. Synthesis of Methyl Betulonate (**7**)

This compound was prepared according to a procedure previously described in the literature using Me_2_SO_4_ as alkylating agent and PCC as oxidant [3]. White solid (882.6 mg, 86% yield). Mp 165-166 °C (Lit. [21] 168 °C). ^1^H NMR (300.13 MHz, CDCl_3_): δ 0.92 (s, 3H, H-25), 0.95 and 0.97 (2 s, 6H, H-26,27), 1.02 and 1.07 (2 s, 6H, H-23,24), 1.15–1.75 (m, 17H), 1.69 (s, 3H, H-30), 1.83–1.94 (m, 3H), 2.17–2.28 (m, 2H), 2.34–2.55 (m, 2H), 2.96–3.04 (m, 1H, H-19), 3.67 (s, 3H, 28-OC*H*_3_), 4.60–4.61 (m, 1H, H-29), 4.74 (d, *J* 2.1 Hz, 1H, H-29) ppm. ^13^C NMR (75.47 MHz, CDCl_3_): δ 14.6, 15.8 and 16.0 (C-25,26,27), 19.4 (C-30), 19.6 (*C*H_2_), 21.1 and 26.6 (C-23,24), 21.4 (*C*H_2_), 25.5 (*C*H_2_), 29.7 (*C*H_2_), 30.6 (*C*H_2_), 32.1 (*C*H_2_), 33.6 (*C*H_2_), 34.2 (*C*H_2_), 36.91*, 36.94 (*C*H_2_), 38.3 (*C*H), 39.6 (*C*H_2_), 40.6*, 42.4*, 46.9 (C-19), 47.4 (C-4), 49.4 (*C*H), 49.9 (*C*H), 51.3 (28-O*C*H_3_), 55.0 (C-5), 56.5*, 109.6 (C-29), 150.5 (C-20), 176.6 (C-28), 218.2 (C-3) ppm. *Unprotonated carbons (C-8,10,14,17). HRMS-ESI *m*/*z* calcd. for [C_31_H_48_O_3_+H]^+^: 469.3682, found: 469.3684.

#### 2.1.3. Synthesis of Methyl (*E*)-2-Benzylidenebetulonate Derivatives **9a**–**d** and **14**, and Methyl (*E*,*E*)-2-[-3-(3,4-Dimethoxyphenyl)allylidene]betulonate (**12**)

NaH (51.3 mg, 2.14 mmol) was added to a solution of methyl betulonate (**7**) (200 mg, 0.427 mmol) in dry THF (8 mL). After few minutes, the appropriate aldehyde **8a**–**d**, **11**, or **13** (0.641 mmol) was added and the reaction mixture was stirred under N_2_ at room temperature overnight. After this period, it was poured onto ice and H_2_O, and the pH was adjusted to 1 with diluted HCl. Then, this aqueous solution was extracted twice with DCM and the organic layer was dried over anhydrous Na_2_SO_4_. The solvent was evaporated to dryness, and the obtained residue was purified by silica gel column chromatography or preparative thin layer chromatography using DCM (for **9a**) and a mixture of Hex/EtOAc [(10:1) for **9b**; (5:1) for **9c**,**d** and **12**; (3:1) for **14**] as eluents.

*Methyl (E)-2-Benzylidenebetulonate* (**9a**). White solid (214 mg, 90% yield). Mp 231–232 °C. ^1^H NMR (300.13 MHz, CDCl_3_): δ 0.79 (s, 3H, H-25), 0.96 and 1.01 (2 s, 6H, H-26,27), 1.12 and 1.15 (2 s, 6H, H-23,24), 1.72 (s, 3H, H-30), 0.71–1.96 (m, 18H), 2.17–2.31 (m, 3H, H-1 + 2 CH), 2.97–3.09 (m, 2H, H-1 + CH), 3.68 (s, 3H, 28-OCH_3_), 4.65 (dd, J 1.4 and 2.4 Hz, 1H, H-29), 4.76 (d, J 2.4 Hz, 1H, H-29), 7.29–7.44 (m, 5H, H-2′,3′,4′,5′,6′), 7.48–7.49 (m, 1H, –CH=) ppm. ^13^C NMR (75.47 MHz, CDCl_3_): δ 14.6 and 15.4 (C-26,27), 15.8 (C-25), 19.5 (C-30), 20.4 (CH_2_), 21.7 (CH_2_), 22.4 and 29.5 (C-23,24), 25.7 (CH_2_), 29.7 (CH_2_), 30.7 (CH_2_), 32.1 (CH_2_), 33.1 (CH_2_), 36.5 (C-10), 36.9 (CH_2_), 38.3 (CH), 40.5*, 42.5*, 44.4 (C-1), 45.2 (C-4), 46.9 (CH), 48.5 (CH), 49.4 (CH), 51.3 (28-OCH_3_), 52.8 (C-5), 56.6*, 109.5 (C-29), 128.4 (C-4′), 128.5 (C-3′,5′), 130.3 (C-2′,6′), 134.3 (C-2), 136.0 (C-1′), 137.3 (–CH=), 150.8 (C-20), 176.6 (C-28), 208.3 (C-3) ppm. *Unprotonated carbons (C-8,14,17). HRMS-ESI *m*/*z* calcd. for [C_38_H_52_O_3_+H]^+^: 557.3995, found: 557.4001.

*Methyl (E)-2-(4-Methoxybenzylidene)betulonate* (**9b**). White solid (215 mg, 86% yield). Mp 133–135 °C. *R = (E)-4-methoxybenzilidene:* ^1^H NMR (300.13 MHz, CDCl_3_): δ 3.85 (s, 3H, 4′-OC*H*_3_), 6.95 (d, *J* 8.8 Hz, 2H, H-3′,5′), 7.41 (d, *J* 8.8 Hz, 2H, H-2′,6′), 7.46 (br s, 1H, –C*H*=) ppm. ^13^C NMR (75.47 MHz, CDCl_3_): δ 55.3 (4′-O*C*H_3_), 114.0 (C-3′,5′), 128.7 (C-1′), 132.2 (C-2′,6′), 137.2 (−*C*H=), 159.8 (C-4′) ppm. HRMS-ESI *m*/*z* calcd. for [C_39_H_54_O_4_+H]^+^: 587.4100, found: 587.4103.

*Methyl (E)-2-(3,4-Dimethoxybenzylidene)betulonate* (**9c**). Light yellow solid (187 mg, 71% yield). Mp 197–198 °C. *R = (E)-3,4-dimethoxybenzylidene:* ^1^H NMR (300.13 MHz, CDCl_3_): δ 3.89 and 3.93 [2 s, 6H, 3′,4′-(OC*H*_3_)_2_], 6.94 (d, *J* 8.4 Hz, 1H, H-5′), 6.94 (d, *J* 2.4 Hz, 1H, H-2′), 7.07 (dd, *J* 2.4 and 8.4 Hz, 1H, H-6′), 7.44 (br s, 1H, –C*H*=) ppm. ^13^C NMR (75.47 MHz, CDCl_3_): δ 55.88 and 55.92 [3′,4′-(O*C*H_3_)_2_], 111.0 (C-5′), 114.3 (C-2′), 123.0 (C-6′), 128.9 (C-1′), 137.3 (−*C*H=), 148.6 (C-3′), 149.4 (C-4′) ppm. HRMS-ESI *m*/*z* calcd. for [C_40_H_56_O_5_+H]^+^: 617.4206, found: 617.4210.

*Methyl (E)-2-(3,4,5-Trimethoxybenzylidene)betulonate* (**9d**). Light yellow solid (199 mg, 72% yield). Mp 183–184 °C. *R = (E)-3,4,5-trimethoxybenzylidene:* ^1^H NMR (300.13 MHz, CDCl_3_): δ 3.86 and 3.89 [2 s, 9H, 3′,4′,5′-(OC*H*_3_)_3_], 6.64 (s, 2H, H-2′,6′), 7.40 (br s, 1H, –C*H*=) ppm. ^13^C NMR (75.47 MHz, CDCl_3_): δ 56.2 and 61.0 [3′,4′,5′-(O*C*H_3_)_3_], 107.7 (C-2′,6′), 131.5 (C-1′), 137.3 (−*C*H=), 138.5 and 152.9 (C-3′,4′,5′) ppm. HRMS-ESI *m*/*z* calcd. for [C_41_H_58_O_6_+H]^+^: 647.4312, found: 617.4305.

*Methyl (E,E)-2-[3-(3,4-Dimethoxyphenyl)allylidene]betulonate* (**12**). Light yellow solid (148 mg, 54% yield). *R = (E,E)-3-(3,4-dimethoxyphenyl)allylidene:* ^1^H NMR (300.13 MHz, CDCl_3_): δ 3.91 (s, 3H, 4″-OC*H*_3_), 3.96 (s, 3H, 3″-OC*H*_3_), 6.77–6.89 (m, 1H, H-3′), 6.78 (dd, *J* 11.2 and 26.9 Hz, 1H, H-2′), 6.86 (d, *J* 8.5 Hz, 1H, H-5″), 6.98 (d, *J* 1.9 Hz, 1H, H-2″), 7.09 (dd, *J* 1.9 and 8.5 Hz, 1H, H-6″), 7.23 (d, *J* 11.2 Hz, 1H, H-1′) ppm. ^13^C NMR (75.47 MHz, CDCl_3_): δ 56.0 (4″-O*C*H_3_), 56.2 (3″-O*C*H_3_), 109.9 (C-2″), 111.2 (C-5″), 120.8 (C-6″), 121.5 (C-2′), 129.9 (C-1″), 137.8 (C-1′), 140.9 (C-3′), 149.1 (C-3″), 150.0 (C-4″) ppm. HRMS-ESI *m*/*z* calcd. for [C_42_H_58_O_5_+H]^+^: 643.4362, found: 643.4376.

*Methyl (E)-2-[3,4-Bis(methoxymethoxy)benzylidene]betulonate* (**14**). White solid (179 mg, 62% yield). Mp 99–101 °C. *R = (E)-3,4-bis(methoxymethoxy)benzylidene:* ^1^H NMR (500.13 MHz, CDCl_3_): δ 3.50 (s, 3H, 3′-OCH_2_OC*H*_3_), 3.53 (s, 3H, 4′-OCH_2_OC*H*_3_), 5.21–5.26 (m, 2H, 3′-OC*H*_2_OCH_3_), 5.28 (s, 2H, 4′-OC*H*_2_OCH_3_), 7.08 (dd, *J* 2.1 and 8.5 Hz, 1H, H-6′), 7.19 (d, *J* 8.5 Hz, 1H, H-5′), 7.34 (d, *J* 2.1 Hz, 1H, H-2′), 7.40 (br s, 1H, –C*H*=) ppm. ^13^C NMR (125.77 MHz, CDCl_3_): δ 56.2 and 56.3 (3′,4′-OCH_2_O*C*H_3_), 95.2 (4′-O*C*H_2_OCH_3_), 95.7 (3′-O*C*H_2_OCH_3_), 115.9 (C-5′), 118.8 (C-2′), 125.5 (C-6′), 130.5 (C-1′), 136.9 (−*C*H=), 146.9 (C-3′), 147.5 (C-4′) ppm. HRMS-ESI *m*/*z* calcd. for [C_42_H_60_O_7_+H]^+^: 677.4417, found: 677.4429.

#### 2.1.4. Synthesis of 19,28-Epoxyoleanane-3,28-dione-Derived Polyhydroxylated Compounds **4a**–**d** and **5**

A solution of 1 M BBr_3_ in DCM (1.1 equiv for each protecting group to be cleaved) was slowly added to a cooled solution (ice bath) of methyl (*E*)-2-benzylidenebetulonate derivatives **9a**–**d** or methyl (*E,E*)-2-[3-(3,4-dimethoxyphenyl)allylidene]betulonate (**12**) (100 mg, 0.155–0.180 mmol) in dry DCM (4 mL). The reaction mixture was stirred under N_2_ at room temperature for 1 h. After this period, it was slowly added to H_2_O and vigorously stirred for 1 h. Then, this aqueous solution was extracted twice with EtOAc, and the organic layer was dried over anhydrous Na_2_SO_4_. The solvent was evaporated to dryness, and the obtained residue was purified by preparative thin layer chromatography using a mixture of Hex/EtOAc [(10:1) for **4a** and (2:1) for **4b**] as eluents, or recrystallized from a mixture of EtOAc/Hex (1:10) for compounds **4c**,**d** and **5**.

(*E)-2-Benzylidene-19,28-epoxyoleanane-3,28-dione* (**4a**). White solid (50.8 mg, 52% yield). Mp 268–270 °C. ^1^H NMR (300.13 MHz, CDCl_3_): δ 0.82 (s, 3H, H-25), 0.92 and 0.95 (2 s, 6H, H-26,27), 0.97 and 1.04 (2 s, 6H, H-29,30), 1.13 and 1.16 (2 s, 6H, H-23,24), 0.61–1.93 (m, 20H), 2.26 (dd, *J* 1.7 and 16.3 Hz, 1H, H-1), 3.09 (dd, *J* 1.7 and 16.3 Hz, 1H, H-1), 3.96 (s, 1H, H-19), 7.30–7.45 (m, 5H, H-2′,3′,4′,5′,6′), 7.49–7.51 (m, 1H, –C*H*=) ppm. ^13^C NMR (75.47 MHz, CDCl_3_): δ 13.6 and 15.1 (C-26,27), 16.2 (C-25), 20.2 (*C*H_2_), 21.7 (*C*H_2_), 22.3 and 28.8 (C-23,24), 24.0 (*C*H), 25.5 (*C*H_2_), 26.6 (*C*H_2_), 27.8 (*C*H_2_), 29.5 (*C*H), 31.9 (*C*H_2_), 32.3 (*C*H_2_), 32.5 (*C*H_2_), 33.6*, 36.1 (*C*H), 36.6*, 40.0*, 40.4*, 44.7 (C-1), 45.2*, 46.1*, 46.7 (*C*H), 49.2 (*C*H), 53.0 (*C*H), 85.9 (C-19), 128.42 (C-3′,5′), 128.45 and 136.0 (C-1′,4′), 130.3 (C-2′,6′), 134.1 (C-2), 137.4 (−*C*H=), 179.8 (C-28), 208.1 (C-3) ppm. *Unprotonated carbons (C-4,8,10,14,17,20). HRMS-ESI *m*/*z* calcd. for [C_37_H_50_O_3_+H]^+^: 543.3838, found: 543.3838.

*(E)-2-(4-Hydroxybenzylidene)-19,28-epoxyoleanane-3,28-dione* (**4b**). White solid (38.1 mg, 40% yield). Mp 191–193 °C. ^1^H NMR (300.13 MHz, DMSO-*d*_6_): δ 0.72 (s, 3H, H-25), 0.87 and 0.92 (2 s, 6H, H-26,27), 0.95 and 0.96 (2 s, 6H, H-29,30), 1.02 and 1.05 (2 s, 6H, H-23,24), 0.65–1.92 (m, 20H), 2.37 (d, *J* 16.5 Hz, 1H, H-1), 2.95 (d, *J* 16.5 Hz, 1H, H-1), 4.03 (s, 1H, H-19), 6.83 (d, *J* 8.7 Hz, 2H, H-3′,5′), 7.30 (br s, 1H, –C*H*=), 7.39 (d, *J* 8.7 Hz, 2H, H-2′,6′), 9.95 (s, 1H, 4′-O*H*) ppm. ^13^C NMR (75.47 MHz, DMSO-*d*_6_): δ 13.4 and 14.6 (C-26,27), 15.9 (C-25), 19.8 (*C*H_2_), 20.8*, 21.1 (*C*H_2_), 22.1 and 29.3 (C-23,24), 23.3 and 28.5 (C-29,30), 25.1 (*C*H_2_), 25.9 (*C*H_2_), 27.5 (*C*H_2_), 30.9 (*C*H_2_), 31.9 (*C*H_2_), 32.1 (*C*H_2_), 33.2*, 35.8*, 36.0 (*C*H), 44.1 (C-1), 44.2*, 45.5*, 45.6 (C-18), 48.1 (*C*H), 51.7 (*C*H), 59.8*, 85.1 (C-19), 115.6 (C-3′,5′), 126.4 (C-1′), 130.6 (C-2), 132.5 (C-2′,6′), 136.8 (−*C*H=), 158.3 (C-4′), 179.0 (C-28), 206.2 (C-3) ppm. *Unprotonated carbons (C-4,8,10,14,17,20). HRMS-ESI *m*/*z* calcd. for [C_37_H_50_O_4_+H]^+^: 559.3787, found: 559.3786.

*(E)-2-(3,4-Dihydroxybenzylidene)-19,28-epoxyoleanane-3,28-dione* (**4c**). White solid (51.2 mg, 55% yield). Mp 219–221 °C. ^1^H NMR (500.13 MHz, Acetone-*d*_6_): δ 0.84 (s, 3H, H-25), 1.00 and 1.011 (2 s, 6H, H-26,27), 1.014 and 1.05 (2 s, 6H, H-29,30), 1.09 and 1.14 (2 s, 6H, H-23,24), 0.67–2.02 (m, 20H), 2.40 (d, *J* 16.3 Hz, 1H, H-1), 3.14 (d, *J* 16.3 Hz, 1H, H-1), 4.03 (s, 1H, H-19), 6.90 (d, *J* 8.2 Hz, 1H, H-5′), 6.97 (dd, *J* 2.2 and 8.2 Hz, 1H, H-6′), 7.08 (d, *J* 2.2 Hz, 1H, H-2′), 7.34 (br s, 1H, –C*H*=), 8.21 and 8.42 [2 s, 2H, 3′,4′-(O*H*)_2_] ppm. ^13^C NMR (125.77 MHz, Acetone-*d*_6_): δ 13.9 and 15.3 (C-26,27), 16.5 (C-25), 20.9 (*C*H_2_), 22.4 (*C*H_2_), 26.3 (*C*H_2_), 27.2 (*C*H_2_), 28.6 (*C*H_2_), 23.9 and 29.0 (C-29,30), 22.6 and 29.8** (C-23,24), 32.3 (*C*H_2_), 33.2 (*C*H_2_), 34.2*, 37.1 (C-10), 37.2 (*C*H), 40.8*, 41.0*, 45.3*, 45.5 (C-1), 46.6*, 47.1 (*C*H), 49.7 (C-9), 53.3 (C-5), 86.1 (C-19), 116.2 (C-5′), 118.3 (C-2′), 124.5 (C-6′), 129.0 (C-1′), 132.2 (C-2), 137.9 (–*C*H=), 145.8 (C-3′), 146.8 (C-4′), 179.4 (C-28), 206.8 (C-3) ppm. *Unprotonated carbons (C-4,8,14,17,20). **Overlapped with the solvent residual signal. HRMS-ESI *m*/*z* calcd. for [C_37_H_50_O_5_+H]^+^: 575.3736, found: 575.3743.

*(E)-2-(3,4,5-Trihydroxybenzylidene)-19,28-epoxyoleanane-3,28-dione* (**4d**). Dark brown solid (74.9 mg, 82% yield). Mp > 300 °C. ^1^H NMR (500.13 MHz, Acetone-*d*_6_): δ 0.84 (s, 3H, H-25), 0.997 and 1.009 (2 s, 6H, H-26,27), 1.013 and 1.05 (2 s, 6H, H-29,30), 1.08 and 1.13 (2 s, 6H, H-23,24), 0.65–2.02 (m, 20H), 2.36 (d, *J* 16.4 Hz, 1H, H-1), 3.15 (dd, *J* 1.7 and 16.4 Hz, 1H, H-1), 4.04 (s, 1H, H-19), 6.66 (s, 2H, H-2′,6′), 7.26 (br s, 1H, –C*H*=), 7.82 and 8.17 [2 br s, 3H, 3′,4′,5′-(O*H*)_3_] ppm. ^13^C NMR (125.77 MHz, Acetone-*d*_6_): δ 13.9 and 15.3 (C-26,27), 16.5 (C-25), 20.9 (*C*H_2_), 22.4 (*C*H_2_), 26.3 (*C*H_2_), 27.2 (*C*H_2_), 28.6 (*C*H_2_), 23.9 and 29.0 (C-29,30), 22.6 and 29.8** (C-23,24), 32.3 (*C*H_2_), 33.2 (*C*H_2_), 34.2*, 37.1 (C-10), 37.2 (*C*H), 40.7*, 41.0*, 45.3*, 45.5 (C-1), 46.6*, 47.1 (*C*H), 49.8 (C-9), 53.3 (C-5), 86.1 (C-19), 111.0 (C-2′,6′), 128.0 (C-1′), 132.3 (C-2), 138.2 (−*C*H=), 135.0, 135.1 and 146.3 (C-3′,4′,5′), 179.4 (C-28), 206.8 (C-3) ppm. *Unprotonated carbons (C-4,8,14,17,20). **Overlapped with the residual solvent signal. HRMS-ESI *m*/*z* calcd. for [C_37_H_50_O_6_+H]^+^: 591.3686, found: 591.3874.

*(E,E)-2-[3-(3,4-Dihydroxyphenyl)allylidene]-19,28-epoxyoleanane-3,28-dione* (**5**). Orangish brown solid (51.4 mg, 55% yield). Mp > 300 °C. ^1^H NMR (300.13 MHz, Acetone-*d*_6_): δ 0.87 (s, 3H, H-25), 1.00 and 1.01 (2 s, 6H, H-26,27), 1.02 and 1.05 (2 s, 6H, H-29,30), 1.07 and 1.10 (2 s, 6H, H-23,24), 0.61-2.02 (m, 22H), 2.19–2.26 (m, 1H, H-1), 3.11–3.17 (m, 1H, H-1), 4.04 (s, 1H, H-19), 6.83 (d, *J* 8.2 Hz, 1H, H-5″), 6.86–7.04 (m, 2H, H-2′,3′), 6.97 (dd, *J* 2.1 and 8.2 Hz, 1H, H-6″), 7.12 (d, *J* 2.1 Hz, 1H, H-2″), 7.14–7.18 (m, 1H, H-1′), 8.08 and 8.36 [2 s, 2H, 3″,4″-(O*H*)_2_] ppm. ^13^C NMR (75.47 MHz, Acetone-*d*_6_): δ 13.9 and 15.4 (C-26,27), 16.5 (C-25), 20.9 (*C*H_2_), 22.3 (*C*H_2_), 22.7 and 29.6 (C-23,24), 23.9 and 29.0 (C-29,30), 26.3 (*C*H_2_), 27.2 (*C*H_2_), 28.6 (*C*H_2_), 32.3 (*C*H_2_), 33.2 (*C*H_2_), 33.3 (*C*H_2_), 34.2*, 37.2 (C-10), 40.7*, 41.0*, 43.3 (C-1), 45.3*, 46.6*, 47.1, 49.7 (C-9), 53.7 (C-5), 86.1 (C-19), 114.3 (C-2″), 116.2 (C-5″), 121.1 (C-6″), 121.7 and 141.7 (C-2′,3′), 130.1 (C-1″), 132.5 (C-2), 138.1 (C-1′), 146.1 (C-3″), 147.3 (C-4″), 179.5 (C-28), 206.5 (C-3) ppm. *Unprotonated carbons (C-4,8,14,17,20). **Overlapped with the residual solvent signal. HRMS-ESI *m*/*z* calcd. for [C_39_H_52_O_5_+H]^+^: 601.3893, found: 601.3905.

#### 2.1.5. Synthesis of Methyl (*E*)-2-(3,4-Dihydroxybenzylidene)betulonate (**6**)

A diluted solution of HCl 20% (5 mL) was added to a solution of methyl (*E*)-2-[3,4-bis(methoxymethoxy)benzylidene]betulonate (**14**) (89.7 mg, 0.133 mmol) in THF (2.5 mL). The reaction was stirred under N_2_ at room temperature for 3 h. After this period, the reaction mixture was poured into ice and water. Then, this aqueous solution was extracted twice with EtOAc, and the organic layer was dried over anhydrous Na_2_SO_4_. The solvent was evaporated to dryness, affording the expected methyl (*E*)-2-(3,4-dihydroxybenzylidene)betulonate (**6**) as a white solid (64.0 mg, 82% yield). Mp 170–172 °C. ^1^H NMR (300.13 MHz, CDCl_3_): δ 0.78 (s, 3H, H-25), 0.96 and 1.02 (2 s, 6H, H-26,27), 1.11 and 1.16 (2 s, 6H, H-23,24), 1.73 (s, 3H, H-30), 0.71–1.96 (m, 18H), 2.18-2.32 (m, 3H, H-1 + 2 C*H*), 2.98-3.08 (m, 2H, H-1 + C*H*), 3.68 (s, 3H, 28-OC*H*_3_), 4.66 (s, 1H, H-29), 4.77 (s, 1H, H-29), 5.92 and 7.30 [2 br s, 2H, 3′,4′-(O*H*)_2_], 6.95 (d, *J* 8.5 Hz, 1H, H-5′), 7.00 (d, *J* 8.5 Hz, 1H, H-6′), 7.17 (br s, 1H, H-2′), 7.49 (br s, 1H, –C*H*=) ppm. ^13^C NMR (75.47 MHz, CDCl_3_): δ 14.6 and 15.4 (C-26,27), 15.9 (C-25), 19.5 (C-30), 20.4 (*C*H_2_), 21.7 (*C*H_2_), 22.3 and 29.6 (C-23,24), 25.7 (*C*H_2_), 29.7 (*C*H_2_), 30.7 (*C*H_2_), 32.1 (*C*H_2_), 33.0 (*C*H_2_), 36.4 (C-10), 37.0 (*C*H_2_), 38.3 (*C*H), 40.5*, 42.5*, 44.6 (C-1), 45.1 (C-4), 46.9 (*C*H), 48.5 (*C*H), 49.4 (*C*H), 51.4 (28-O*C*H_3_), 52.5 (C-5), 56.6*, 109.6 (C-29), 115.2 (C-5′), 118.8 (C-2′), 123.4 (C-6′), 128.5 (C-1′), 131.6 (C-2), 138.9 (−*C*H=), 143.7 (C-3′), 145.5 (C-4′), 150.7 (C-20), 176.7 (C-28), 210.1 (C-3) ppm. *Unprotonated carbons (C-8,14,17). HRMS-ESI *m*/*z* calcd. for [C_38_H_52_O_5_+H]^+^: 589.3893, found: 589.3906.

### 2.2. DPPH^•^ and ABTS^•+^ Scavenging Assays

The compounds capacity for scavenging DPPH^•^ and ABTS^•+^ was evaluated following the procedures previously described by Catarino et al. [22,23]. The compounds under study as well as the positive controls, α-tocopherol and quercetin, were dissolved in a mixture of EtOH/H_2_O 70%. The DPPH^•^ and ABTS^•+^ stock solutions were prepared using EtOH as solvent, and their absorbance was adjusted between 0.721 ± 0.004 and 0.79 ± 0.01 at 517 and 734 nm, respectively. These assays were undertaken at room temperature. In each assay, three independent experiments were performed, using 5–7 concentrations in duplicate, to obtain the IC_50_ values, which were calculated from the curves of %inhibition vs compound concentration and expressed as mean ± standard error of the mean (SEM).

## 3. Results and Discussion

### 3.1. Synthesis of Polyhydroxylated (E)-2-Benzylidene-19,28-epoxyoleanane-3,28-diones ***4a**–**d***

**Scheme 1:**
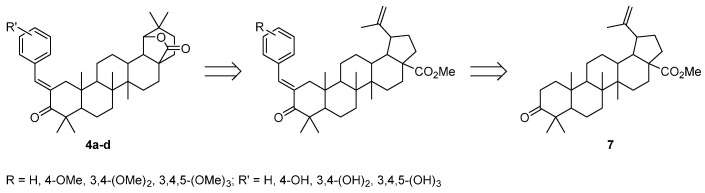
Retrosynthetic analysis of (*E*)-2-benzylidene-19,28-epoxyoleanane-3,28-diones **4a**–**d**.

Polyhydroxylated (*E*)-2-benzylidene-19,28-epoxyoleanane-3,28-diones **4a**–**d** were synthesized in a two-step approach, starting from methyl betulonate (**7**) and involving base-promoted aldol condensations with different benzaldehydes, followed by cleavage of the methyl protecting groups (Scheme 1).

**Scheme 2:**
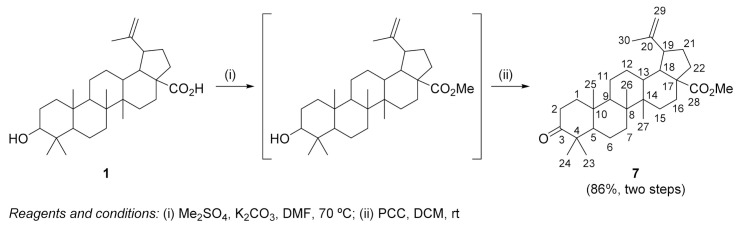
Synthesis of methyl betulonate (**7**).

Firstly, methyl betulonate (**7**) was prepared in very good global yield (86%) from BA (**1**) through the esterification of its carboxyl group with dimethyl sulfate (Me_2_SO_4_), followed by the oxidation of its 3-hydroxy group with pyridinium chlorochromate (PCC) (Scheme 2).

**Scheme 3:**
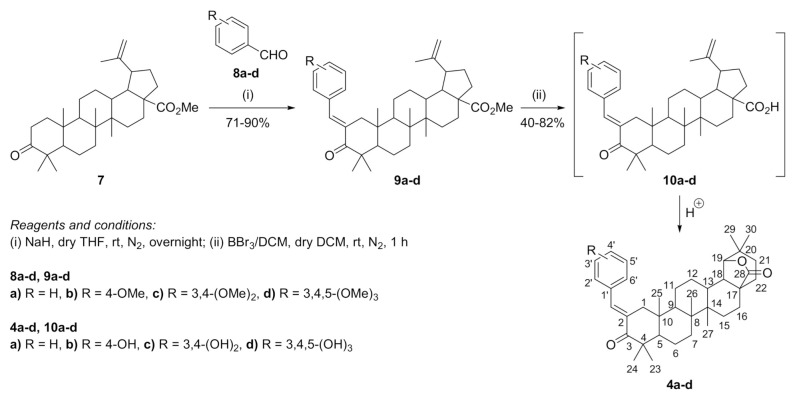
Synthesis of (*E*)-2-benzylidene-19,28-epoxyoleanane-3,28-dione-derived polyhydroxylated compounds **4a**–**d**.

The following step involved the base-promoted aldol condensation of methyl betulonate (**7**) with benzaldehydes **8a**–**d** using sodium hydride as base (Scheme 3), affording methyl (*E*)-2-benzylidenebetulonate derivatives **9a**–**d** in good to excellent yields (71–90%). The main feature in the ^1^H NMR spectra of these compounds is the resonance corresponding to the methylidyne proton of their characteristic α,β-unsaturated carbonyl moiety, which appears as a multiplet at δ_H_ 7.48–7.49 ppm for **9a** or as a broad singlet at δ_H_ 7.40–7.46 ppm for **9b**–**d**. The appearance of this resonance at the aromatic region of ^1^H NMR spectra proves that the aldol condensation reactions were successfully performed. In the case of **9b**–**d**, additional singlets at δ_H_ 3.85–3.93 ppm were observed, which are attributed to the resonances of protons from protecting groups (–OC*H*_3_).

**Scheme 4:**
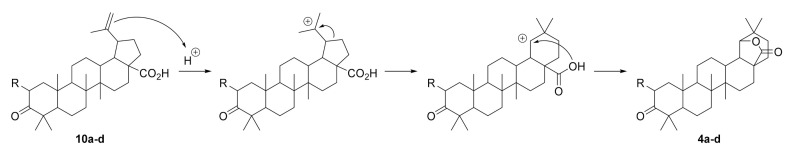
Rearrangement of betulonic acid-derived intermediates **10a**–**d** (not observed) to 19,28-epoxyoleanane-3,28-dione-type compounds **4a**–**d**.

Finally, the last step involved the cleavage of the methyl groups of compounds **9a**–**d** with boron tribromide (BBr_3_), giving rise to the polyhydroxylated (*E*)-2-benzylidene-19,28-epoxyoleanane-3,28-dione derivatives **4a**–**d** in fair to very good yields (40–82%) (Scheme 3). Surprisingly, these compounds were obtained instead of the expected betulonic acid-derived compounds **10a**–**d**, through the acid-catalyzed rearrangement of their E-ring into an oleanane-type skeleton corresponding to derivatives **4a**–**d** (Scheme 4) [24].

This rearrangement takes place during the work-up of the cleavage reactions with water, due to HBr release, and it was confirmed by ^1^H and ^13^C NMR spectroscopy. Therefore, the disappearance of the resonances of the vinylic protons H-29 was observed together with the appearance of a singlet at δ_H_ 3.96–4.04 ppm corresponding to H-19 in the ^1^H NMR spectra. In addition, the singlet corresponding to protons H-30 of the vinylic methyl group shifts to lower frequency values, and there is the appearance of another singlet corresponding to H-29 of the formed methyl group. Furthermore, in the ^13^C NMR spectra, a new resonance corresponding to C-19 of the new C−O single bond appears at δ_C_ 85.1–86.1 ppm.

Another important feature of the ^1^H NMR spectra of compounds **4b**–**d** is the presence of singlets at high frequency values (δ_H_ 7.82–9.95 ppm) corresponding to the formed hydroxy groups.

The synthesized compounds **4a**–**d** differ in the number and position of the hydroxy groups in order to be screened for their antioxidant activity and stablish a structure–activity relationship (SAR).

### 3.2. Radical Scavenging Activity of Polyhydroxylated (E)-2-Benzylidene-19,28-epoxyoleanane-3,28-diones ***4a**–**d***

The radical scavenging ability of new polyhydroxylated (*E*)-2-benzylidene-19,28-epoxyoleanane-3,28-diones **4a**–**d** was tested against DPPH^•^ and ABTS^•+^. Note that DPPH^•^ and ABTS^•+^ assays are widely used as a preliminary assessment of the antioxidant ability of natural or synthetic molecules, and particularly for amphiphilic compounds such as **4a**–**d**, since these radicals can be solubilized in both aqueous and organic media. α-Tocopherol was chosen as positive control, since the synthesized (*E*)-2-benzylidene-19,28-epoxyoleanane-3,28-diones **4a**–**d** also possess a hydrophobic moiety. Furthermore, some of the synthesized compounds **4a**–**d** present polyphenolic units, so we chose quercetin as positive control too, based on its dietary relevance and acknowledged antioxidant capacities, as well as its wide range of health benefits [25].

Results of the scavenging activity of the tested compounds **4a**–**d** against DPPH^•^ and ABTS^•+^ are summarized in Table 1. These two free radicals were efficiently scavenged by derivatives **4c** and **4d** in a concentration-dependent manner with IC_50_ values varying from 22.1 to 35.1 µM, being **4c** the most efficient scavenger against DPPH^•^, reaching an IC_50_ value of 22.1 ± 0.6 µM, which was even lower than IC_50_ value found for α-tocopherol (24.0 ± 0.4 µM). On the other hand, derivatives **4a** and **4b** did not show any scavenging activity against these two free radicals up to the highest concentration tested (800 µM). Overall, our results are consistent with the theory that the 3,4-dihydroxy (catechol-type) substitution plays an important role in what concerns to the scavenging activity of polyphenolic compounds, and thus, of the (*E*)-2-benzylidene-19,28-epoxyoleanane-3,28-dione polyphenolic derivatives.

### 3.3. Analogues Synthesis of the Hit Compound ***4c*** and Evaluation of Their Radical Scavenging Activity

**Figure 3:**
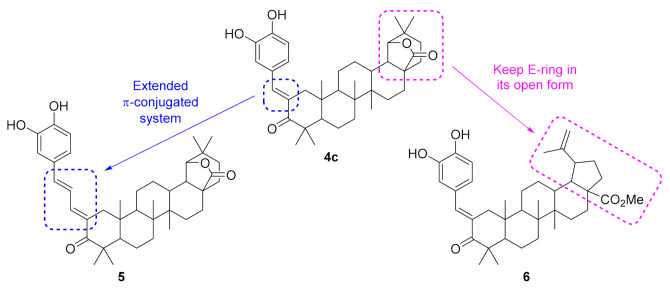
Analogue-based design of compounds **5** and **6**.

Taking into consideration the SAR findings for the synthesized (*E*)-2-benzylidene-19,28-epoxyoleanane-3,28-diones **4a**–**d**, two analogues of the most active compound (derivative **4c**) were synthesized in order to achieve a more complete SAR profile (Figure 3).

**Scheme 5:**
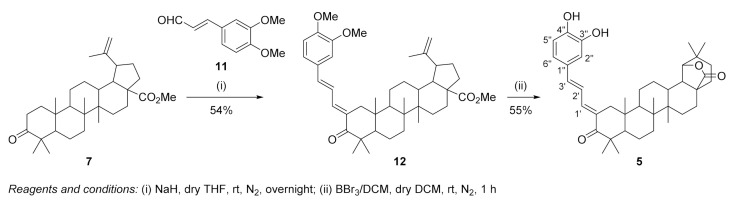
Synthesis of (*E*,*E*)-2-[3-(3,4-hydroxyphenyl)allylidene]-19,28-epoxyoleanane-3,28-dione (**5**).

To begin with, we designed an oleanane-type derivative carrying an extended π-conjugated system at C-2 (Figure 3) to evaluate if this structural feature can contribute to the molecular stabilization and improve the antioxidant activity of this kind of compounds. Thus, using the 3,4-dimethoxycinnamaldehyde (**11**) in the base-promoted aldol condensation reaction with methyl betulonate (**7**), the methyl (*E*,*E*)-2-[-3-(3,4-dimethoxyphenyl)allylidene]betulonate (**12**) was obtained in fair yield (54%) (Scheme 5). Then, after cleavage of its methyl groups using BBr_3_, the (*E*,*E*)-2-[3-(3,4-dihydroxyphenyl)allylidene]-19,28-epoxyoleanane-3,28-dione (**5**) bearing a catechol moiety and an α,β,γ,δ-diunsaturated carbonyl system was also obtained in fair yield (55%).

**Scheme 6:**
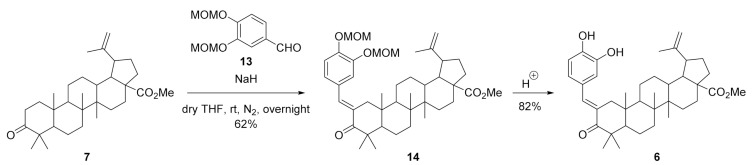
Synthesis of methyl (*E*)-2-(3,4-dihydroxybenzylidene)betulonate (**6**).

With the purpose of comparing the ability of (*E*)-2-benzylidene-19,28-epoxyoleanane-3,28-dione derivative **4c** as antioxidant agent with its correspondent esterified form (Figure 3), methyl (*E*)-2-(3,4-dihydroxybenzylidene)betulonate (**6**) was synthesized through a similar procedure as the one described above. However, instead of using methyl groups as protecting groups, methoxymethyl (MOM) groups were used, since they can be easily cleavage under milder reaction conditions [26], thus preventing the occurrence of the E-ring rearrangement. Compound **6** was obtained in fair global yield (51%) through base-promoted aldol condensation of methyl betulonate (**7**) with 3,4-bis(methoxymethoxy)benzaldehyde (**13**), which had to be previously prepared from 3,4-dihydroxybenzaldehyde, followed by cleavage of the MOM groups using 20% aqueous HCl (Scheme 6).

Regarding the antioxidant capacity of analogues **5** and **6** in comparison with **4c**, the desired effect was not observed in the DPPH^•^ assay, since **4c** was still the most efficient DPPH^•^ scavenger (IC_50_ 22.1 ± 0.6 µM) in comparison with **5** (IC_50_ 24.6 ± 0.6 µM) and **6** (IC_50_ 25.1 ± 0.1 µM) (Table 1). Nonetheless, both analogues **5** and **6** were more efficient ABTS^•+^ scavengers than **4c** as shown by the following order of potencies **5** > **6** ≈ α-tocopherol > **4c** (Table 1). In particular, analogue **5** was the most efficient ABTS^•+^ scavenger (IC_50_ 15.9 ± 0.2 µM), proving that the presence of an extended π-system seems to contribute to the improvement of the scavenging effect of 19,28-epoxyoleanane-3,28-dione-type compounds. In fact, this compound was also more efficient than positive control α-tocopherol (IC_50_ 19.2 ± 0.1 µM) in the ABTS^•+^ assay.

## 4. Conclusions

To sum up, polyhydroxylated 19,28-epoxyoleanane-3,28-dione-type and methyl betulonate-type compounds were synthesized in fair overall yields (30–59%), starting from natural BA (**1**), and were screened as antioxidant agents against DPPH^•^ and ABTS^•+^. Compound **5**, bearing a catechol moiety and an extended π-conjugated carbonyl system, emerged as lead compound, since it revealed to be the most efficient scavenger for ABTS^•+^. In fact, it was more active than α-tocopherol used as positive control and the pristine BA (**1**), which shows its potential to develop a potent amphiphilic antioxidant. Therefore, this study contributed to the valorization of a natural constituent of biomass by-products, since we were able to transform it into new added-value compounds with potential as amphiphilic antioxidants. Their applications can go through the dietary supplementation, food, and skin care industries.

## Figures and Tables

**Table 1:** Scavenging activity of the tested compounds against DPPH^•^ and ABTS^•+^ (IC_50_, mean ± SEM).

Compound	R	IC_50_ (µM)	
		DPPH^•^	ABTS^•+^
Precursors			
BA (**1**)	—	NA^800^ ^μM^	NA^800^ ^μM^
**7**	—	NA^800^ ^μM^	NA^800^ ^μM^
Compounds **4a**–**d** (Section 3.1)			
**4a**	H	NA^800^ ^μM^	NA^800^ ^μM^
**4b**	4-OH	NA^800^ ^μM^	NA^800^ ^μM^
**4c**	3,4-(OH)_2_	22.1 ± 0.6	29.8 ± 0.1
**4d**	3,4,5-(OH)_3_	27.5 ± 0.4	35.1 ± 0.3
Hit **4c** analogues (Section 3.3)			
**5**	—	24.6 ± 0.6	15.9 ± 0.2
**6**	—	25.1 ± 0.1	19.1 ± 0.3
Positive controls			
α-Tocopherol	—	24.0 ± 0.4	19.2 ± 0.1
Quercetin	—	11.4 ± 0.9	5.4 ± 0.1

NA—No activity was found up to the highest tested concentration (in superscript).

## Data Availability

Not applicable.

## Supplementary Materials

The following are available online at https://www.mdpi.com/2076-3921/10/2/148/s1, Figure S1: Structures and numbering system of intermediates **9a**–**d**, **12**, and **14**. Figures S2–S25: ^1^H and ^13^C NMR spectra of all synthesized compounds.
