# Supplementary material for: Functionalization of Betulinic Acid with Polyphenolic Fragments for the Development of New Amphiphilic Antioxidants
Source: Antioxidants (Basel). 2021 Jan 20;10(2):148. doi: 10.3390/antiox10020148 (PMC7909560; doi:10.3390/antiox10020148)
Supplement: Supplementary file 1 [file antioxidants-10-00148-s001.pdf]

Functionalization of Betulinic Acid with Polyphenolic Fragments for the Development of New Amphiphilic Antioxidants

Joana L. C. Sousa [1,2,*], Cristiana Gonçalves [1], Ricardo M. Ferreira [1], Susana M. Cardoso [1], Carmen S. R. Freire [2], Armando J. D. Silvestre [2] and Artur M. S. Silva [1,*]

[1] LAQV-REQUIMTE, Department of Chemistry, University of Aveiro, 3810-193 Aveiro, Portugal; csng@ua.pt (C.G.); ric.ferreira@ua.pt (R.M.F.); susanacardoso@ua.pt (S.M.C.)

[2] CICECO – Aveiro Institute of Materials, Department of Chemistry, University of Aveiro, 3810-193 Aveiro, Portugal; cfreire@ua.pt (C.S.R.F.); armsil@ua.pt (A.J.D.S.)

* Correspondence: joanasousa@ua.pt (J.L.C.S.); artur.silva@ua.pt (A.M.S.S.); Tel.: +351-234-370-714 (A.M.S.S.)

Contents

Figure S1. Structures and numbering system of methyl (*E*)-2-benzylidenebetulonate derivatives **9a-d** and **14**, and methyl (*E*,*E*)-2-allylidenebetulonate derivative **12**. 3

Figure S2. ^{1}H NMR spectrum of methyl (*E*)-2-benzylidenebetulonate (**9a**) (300.13 MHz, $CDCl_3$). 4

Figure S3. ^{13}C NMR spectrum of methyl (*E*)-2-benzylidenebetulonate (**9a**) (75.47 MHz, $CDCl_3$). 4

Figure S4. ^{1}H NMR spectrum of methyl (*E*)-2-(4-methoxybenzylidene)betulonate (**9b**) (300.13 MHz, $CDCl_3$). 5

Figure S5. ^{13}C NMR spectrum of methyl (*E*)-2-(4-methoxybenzylidene)betulonate (**9b**) (75.47 MHz, $CDCl_3$). 5

Figure S6. ^{1}H NMR spectrum of methyl (*E*)-2-(3,4-dimethoxybenzylidene)betulonate (**9c**) (300.13 MHz, $CDCl_3$). 6

Figure S7. ^{13}C NMR spectrum of methyl (*E*)-2-(3,4-dimethoxybenzylidene)betulonate (**9c**) (75.47 MHz, $CDCl_3$). 6

Figure S8. ^{1}H NMR spectrum of methyl (*E*)-2-(3,4,5-trimethoxybenzylidene)betulonate (**9d**) (300.13 MHz, $CDCl_3$). 7

Figure S9. ^{13}C NMR spectrum of methyl (*E*)-2-(3,4,5-trimethoxybenzylidene)betulonate (**9d**) (75.47 MHz, $CDCl_3$). 7

Figure S10. ^{1}H NMR spectrum of methyl (*E*,*E*)-2-[3-(3,4-dimethoxyphenyl)allylidene]betulonate (**12**) (300.13 MHz, $CDCl_3$). 8

Figure S11. ^{13}C NMR spectrum of methyl (*E*,*E*)-2-[3-(3,4-dimethoxyphenyl)allylidene]betulonate (**12**) (75.47 MHz, $CDCl_3$). 8

Figure S12. ^{1}H NMR spectrum of methyl (*E*)-2-[3,4-bis(methoxymethoxy)benzylidene]betulonate (**14**) (500.13 MHz, $CDCl_3$). 9
Figure S13. ^{13}C NMR spectrum of methyl (*E*)-2-[3,4-bis(methoxymethoxy)benzylidene]betulonate (**14**) (125.77 MHz, $CDCl_3$). 9
Figure S14. ^{1}H NMR spectrum of (*E*)-2-benzylidene-19,28-epoxyoleanane-3,28-dione (**4a**) (300.13 MHz, $CDCl_3$). 10
Figure S15. ^{13}C NMR spectrum of (*E*)-2-benzylidene-19,28-epoxyoleanane-3,28-dione (**4a**) (75.47 MHz, $CDCl_3$). 10
Figure S16. ^{1}H NMR spectrum of (*E*)-2-(4-hydroxybenzylidene)-19,28-epoxyoleanane-3,28-dione (**4b**) (300.13 MHz, DMSO-d_6). 11
Figure S17. ^{13}C NMR spectrum of (*E*)-2-(4-hydroxybenzylidene)-19,28-epoxyoleanane-3,28-dione (**4b**) (75.47 MHz, DMSO-d_6). 11
Figure S18. ^{1}H NMR spectrum of (*E*)-2-(3,4-dihydroxybenzylidene)-19,28-epoxyoleanane-3,28-dione (**4c**) (500.13 MHz, Acetone-d_6). 12
Figure S19. ^{13}C NMR spectrum of (*E*)-2-(3,4-dihydroxybenzylidene)-19,28-epoxyoleanane-3,28-dione (**4c**) (125.77 MHz, Acetone-d_6). 12
Figure S20. ^{1}H NMR spectrum of (*E*)-2-(3,4,5-trihydroxybenzylidene)-19,28-epoxyoleanane-3,28-dione (**4d**) (500.13 MHz, Acetone-d_6). 13
Figure S21. ^{13}C NMR spectrum of (*E*)-2-(3,4,5-trihydroxybenzylidene)-19,28-epoxyoleanane-3,28-dione (**4d**) (125.77 MHz, Acetone-d_6). 13
Figure S22. ^{1}H NMR spectrum of (*E*,*E*)-2-[3-(3,4-dihydroxyphenyl)allylidene]-19,28-epoxyoleanane-3,28-dione (**5**) (300.13 MHz, Acetone-d_6). 14
Figure S23. ^{13}C NMR spectrum of (*E*,*E*)-2-[3-(3,4-dihydroxyphenyl)allylidene]-19,28-epoxyoleanane-3,28-dione (**5**) (75.47 MHz, Acetone-d_6). 14
Figure S24. ^{1}H NMR spectrum of methyl (*E*)-2-(3,4-dihydroxybenzylidene)betulonate (**6**) (300.13 MHz, $CDCl_3$). 15
Figure S25. ^{13}C NMR spectrum of methyl (*E*)-2-(3,4-dihydroxybenzylidene)betulonate (**6**) (75.47 MHz, $CDCl_3$). 15

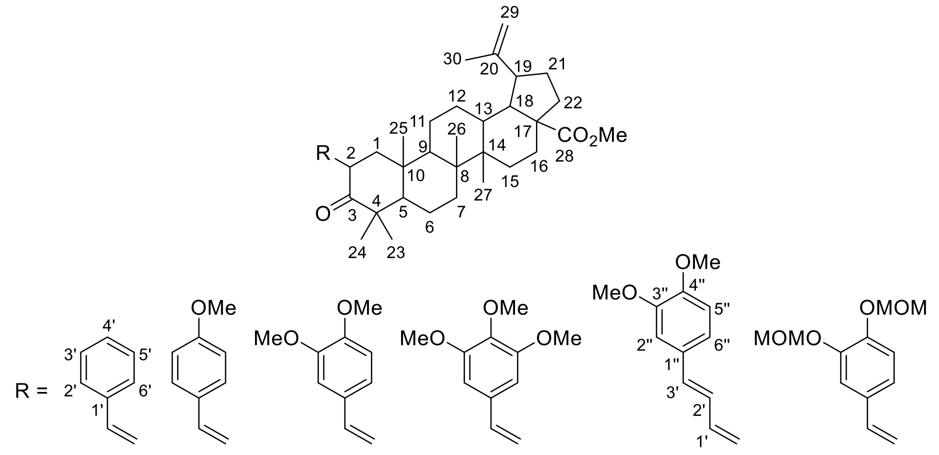

9a **9b** **9c** **9d** **12** **14**

Figure S1. Structures and numbering system of methyl (*E*)-2-benzylidenebetulonate derivatives **9a-d** and **14**, and methyl (*E*,*E*)-2-allylidenebetulonate derivative **12**.

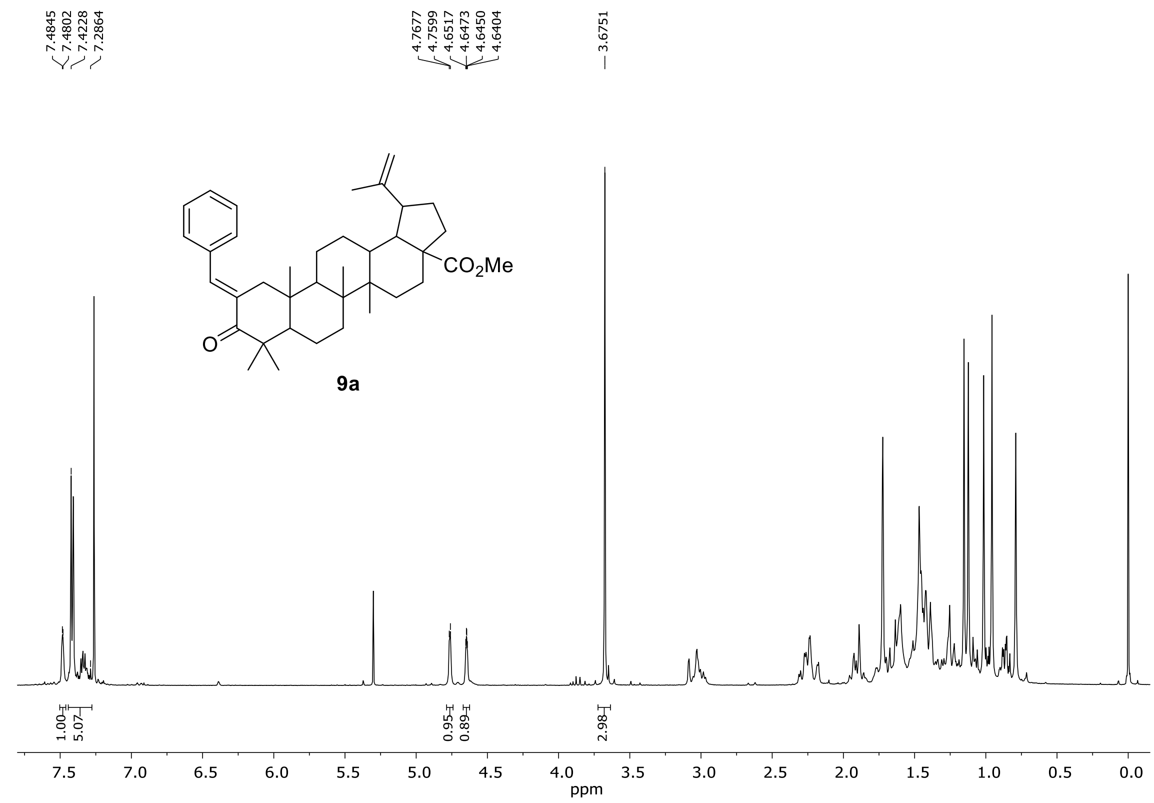

Figure S2. ^{1}H NMR spectrum of methyl (*E*)-2-benzylidenebetulonate (**9a**) (300.13 MHz, $CDCl_3$).

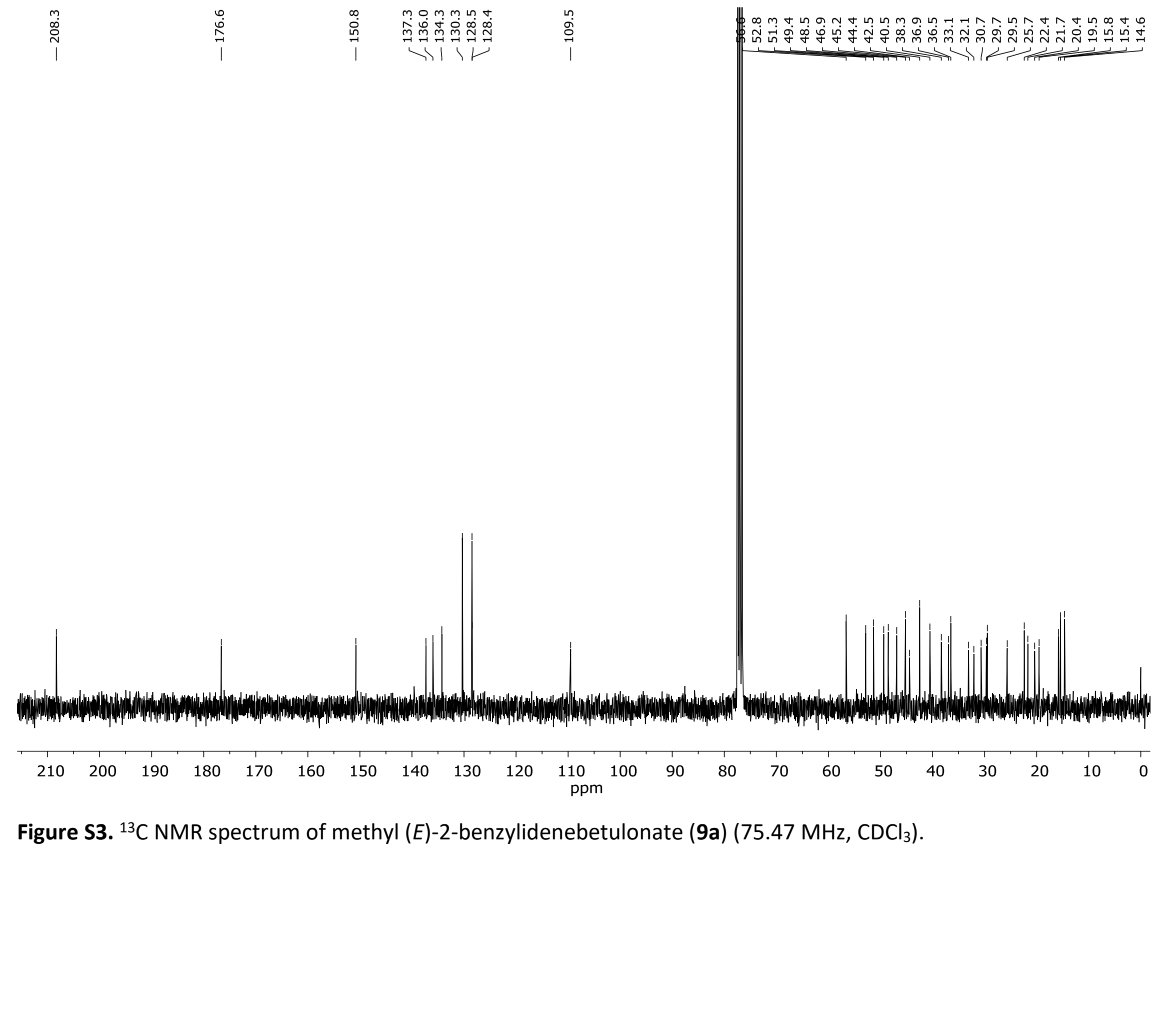

Figure S3. ^{13}C NMR spectrum of methyl (*E*)-2-benzylidenebetulonate (**9a**) (75.47 MHz, $CDCl_3$).

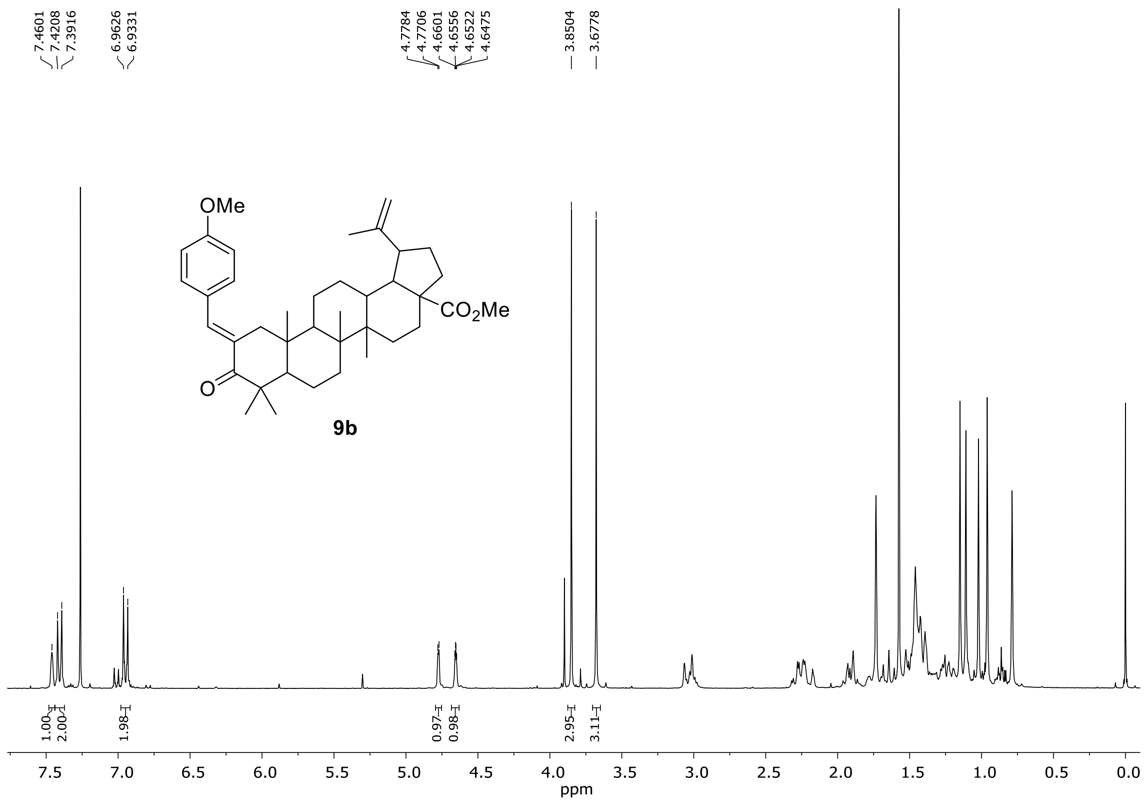

Figure S4. ^{1}H NMR spectrum of methyl (*E*)-2-(4-methoxybenzylidene)betulonate (**9b**) (300.13 MHz, $CDCl_3$).

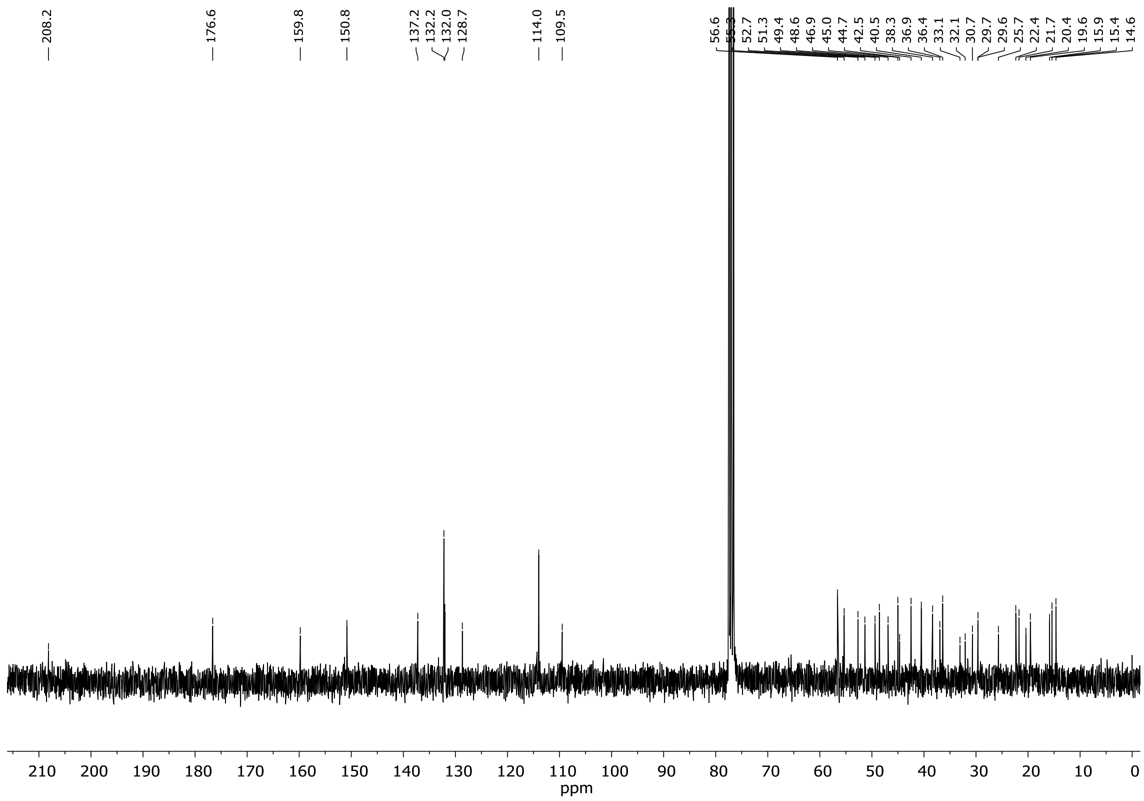

Figure S5. ^{13}C NMR spectrum of methyl (*E*)-2-(4-methoxybenzylidene)betulonate (**9b**) (75.47 MHz, $CDCl_3$).

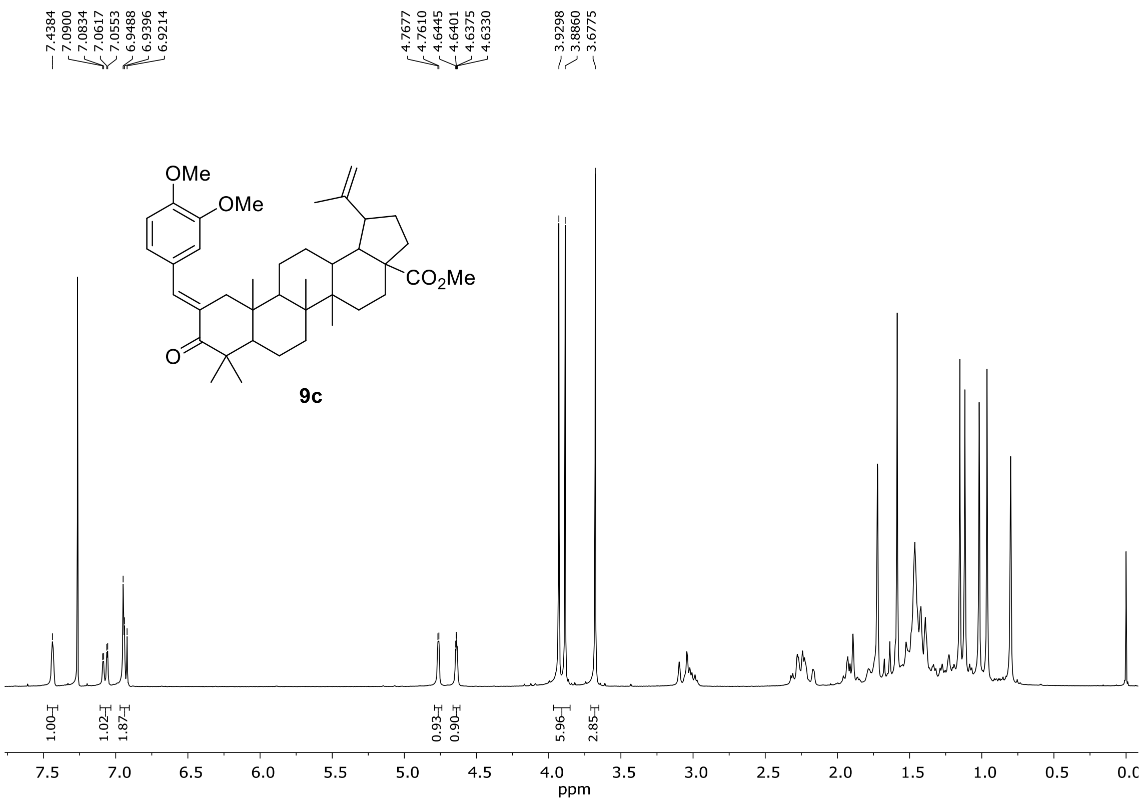

Figure S6. ^{1}H NMR spectrum of methyl (*E*)-2-(3,4-dimethoxybenzylidene)betulonate (**9c**) (300.13 MHz, $CDCl_3$).

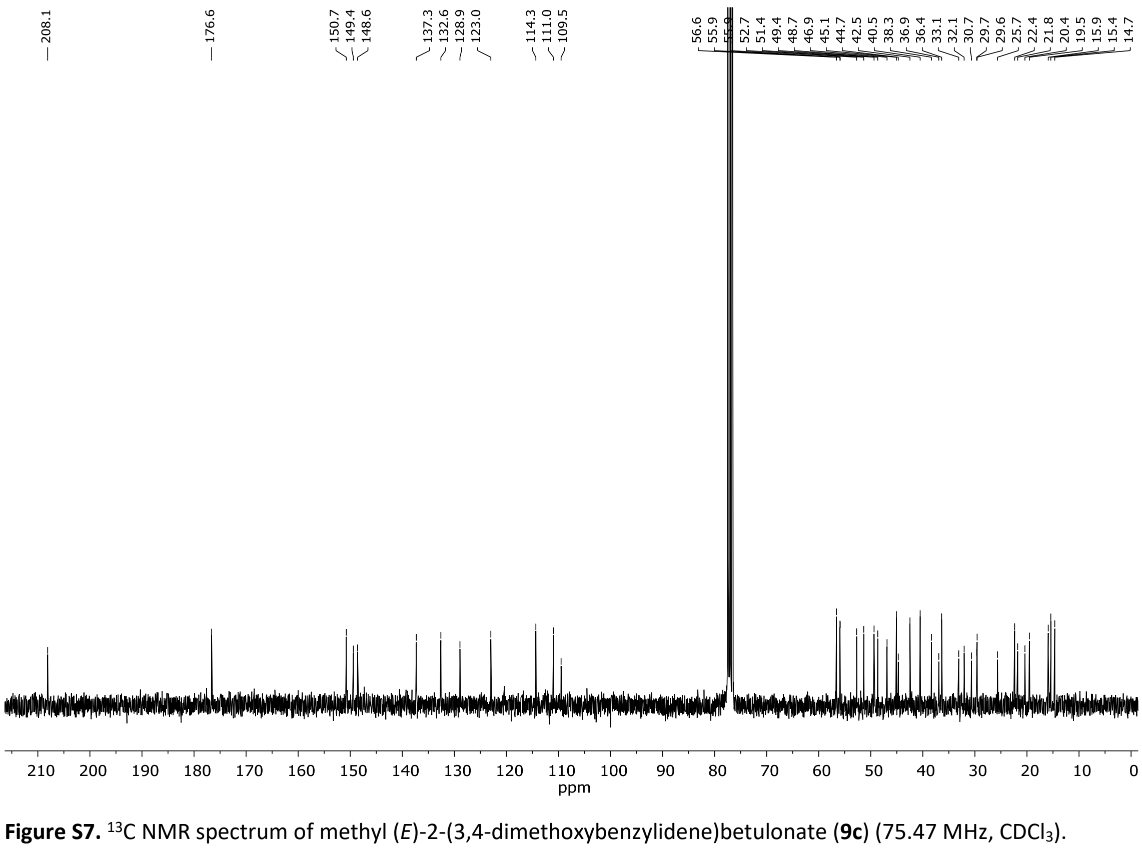

Figure S7. ^{13}C NMR spectrum of methyl (*E*)-2-(3,4-dimethoxybenzylidene)betulonate (**9c**) (75.47 MHz, $CDCl_3$).

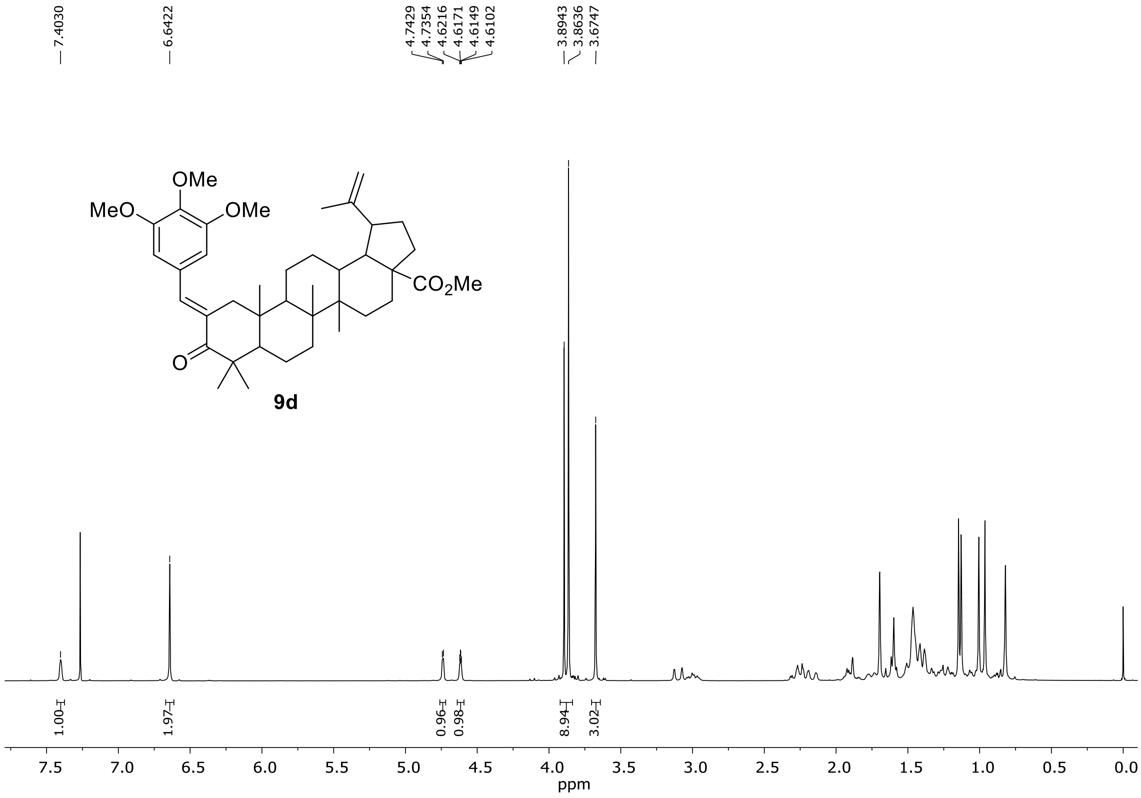

Figure S8. ^{1}H NMR spectrum of methyl (*E*)-2-(3,4,5-trimethoxybenzylidene)betulonate (**9d**) (300.13 MHz, $CDCl_3$).

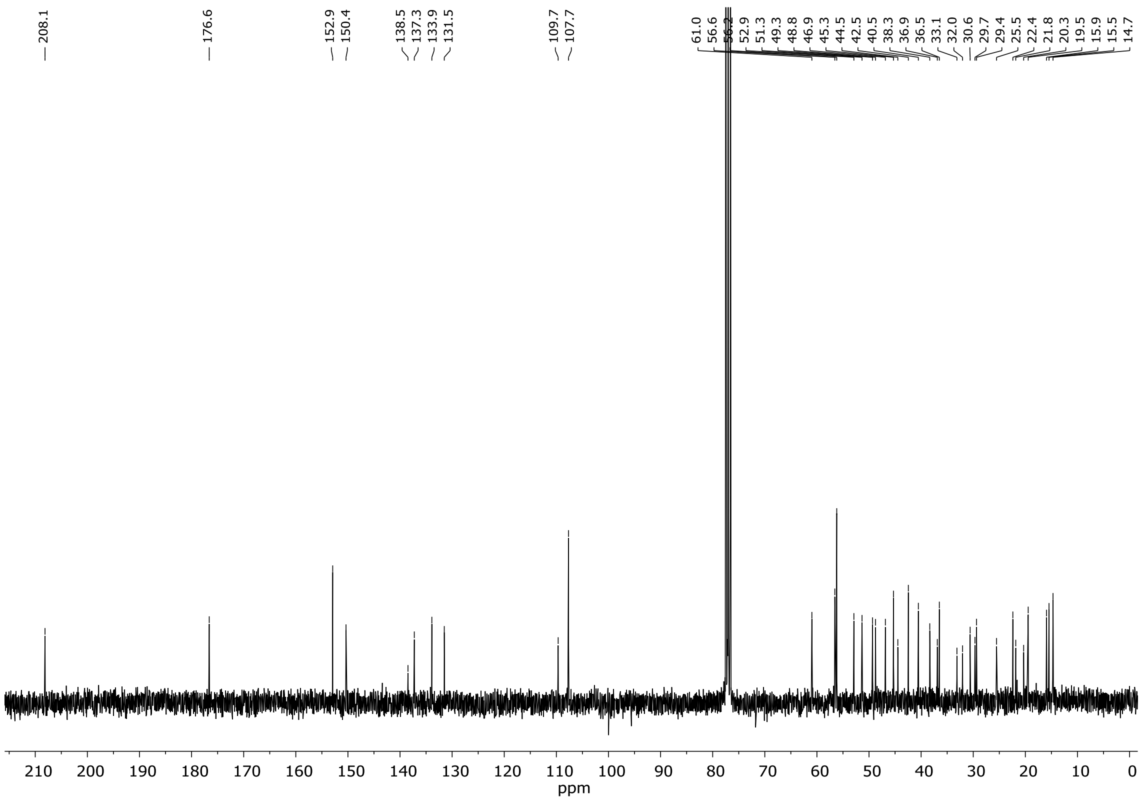

Figure S9. ^{13}C NMR spectrum of methyl (*E*)-2-(3,4,5-trimethoxybenzylidene)betulonate (**9d**) (75.47 MHz, $CDCl_3$).

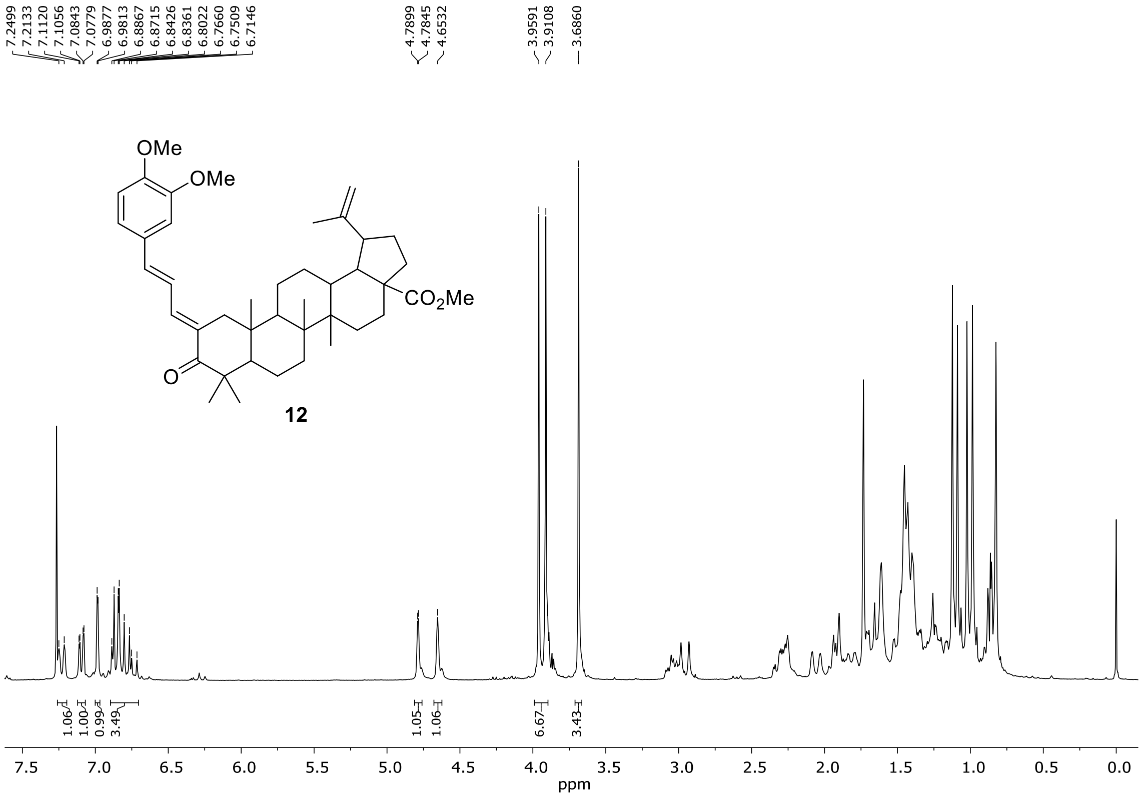

Figure S10. ^{1}H NMR spectrum of methyl (*E*,*E*)-2-[3-(3,4-dimethoxyphenyl)allylidene]betulonate (**12**) (300.13 MHz, $CDCl_3$).

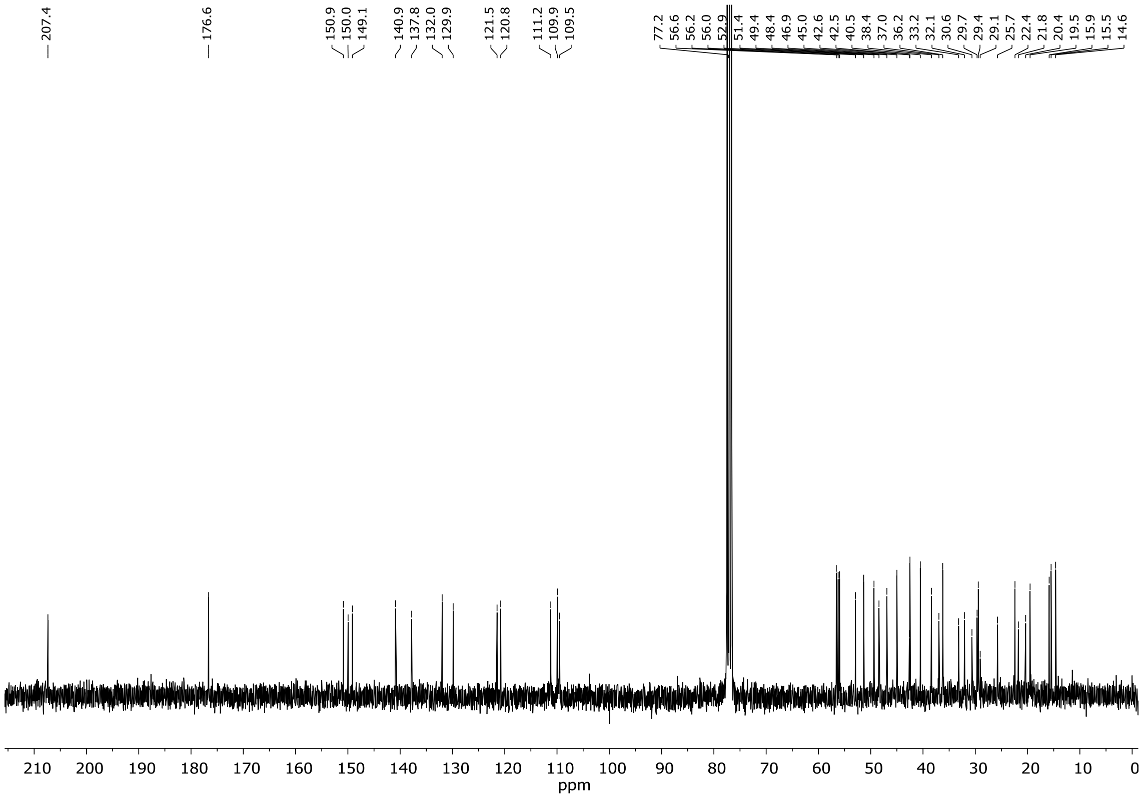

Figure S11. ^{13}C NMR spectrum of methyl (*E*,*E*)-2-[3-(3,4-dimethoxyphenyl)allylidene]betulonate (**12**) (75.47 MHz, $CDCl_3$).

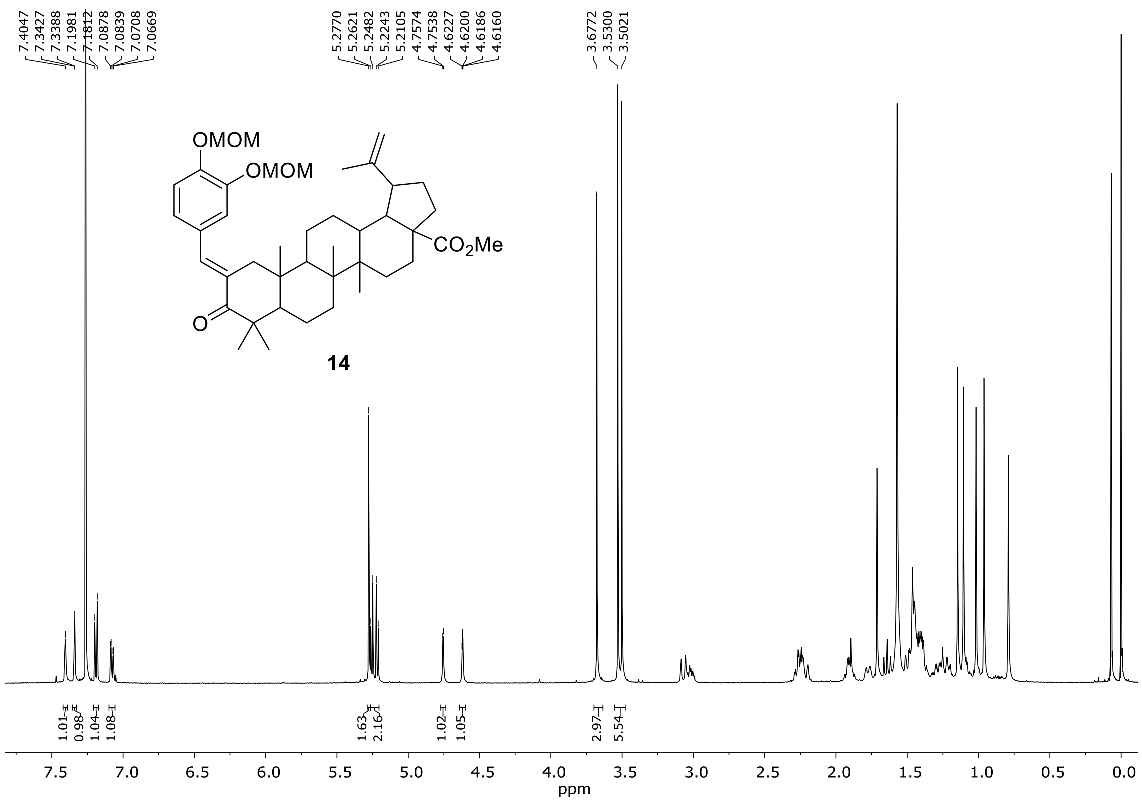

Figure S12. ^{1}H NMR spectrum of methyl (*E*)-2-[3,4-bis(methoxymethoxy)benzylidene]betulonate (**14**) (500.13 MHz, $CDCl_3$).

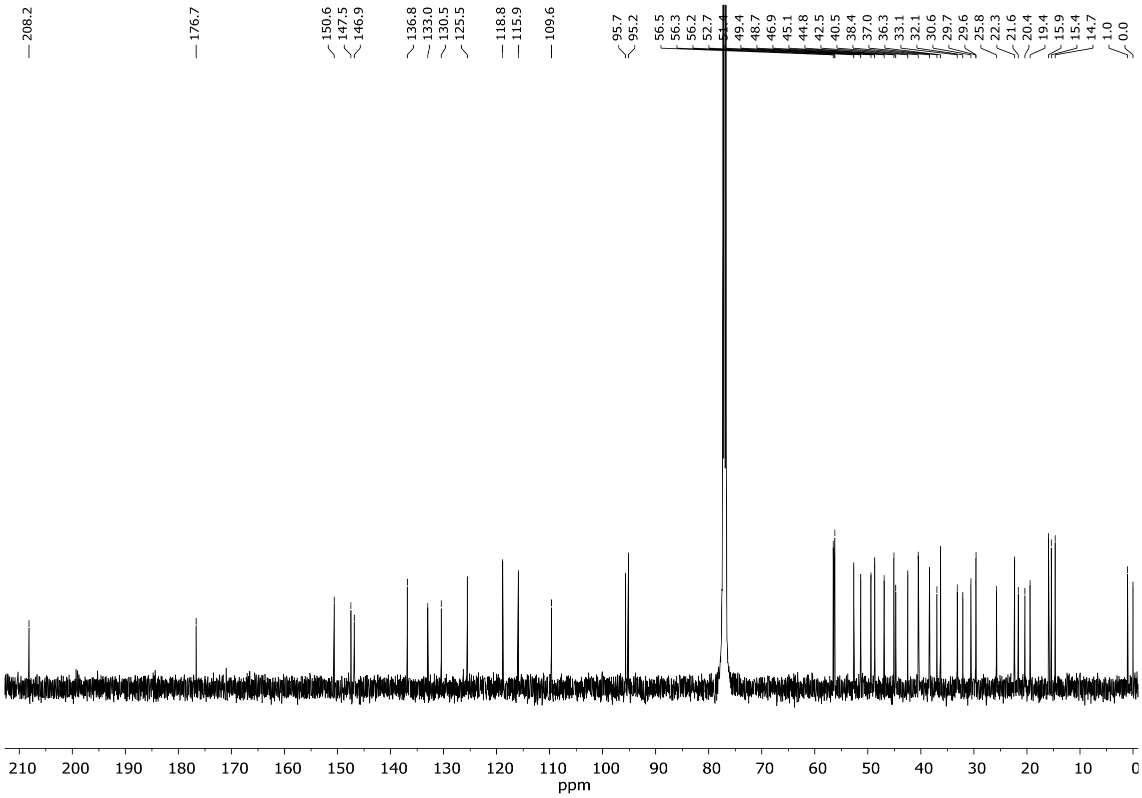

Figure S13. ^{13}C NMR spectrum of methyl (*E*)-2-[3,4-bis(methoxymethoxy)benzylidene]betulonate (**14**) (125.77 MHz, $CDCl_3$).

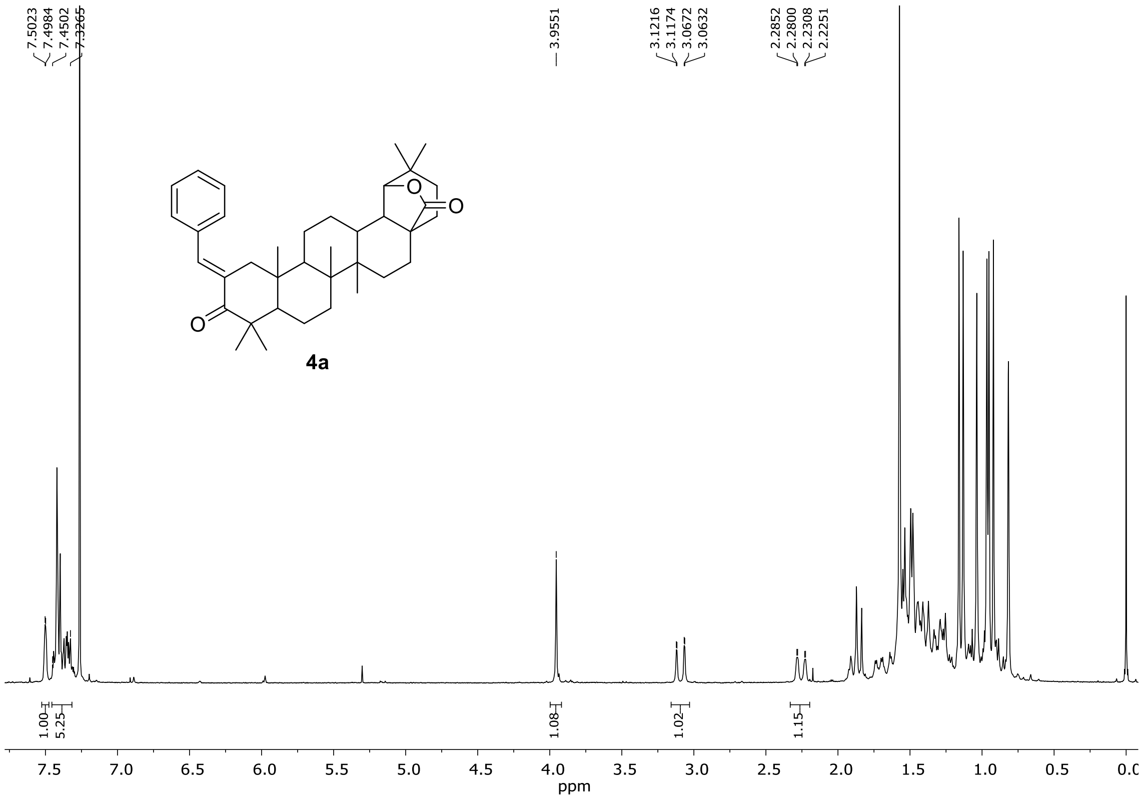

Figure S14. ^{1}H NMR spectrum of (*E*)-2-benzylidene-19,28-epoxyoleanane-3,28-dione (**4a**) (300.13 MHz, $CDCl_3$).

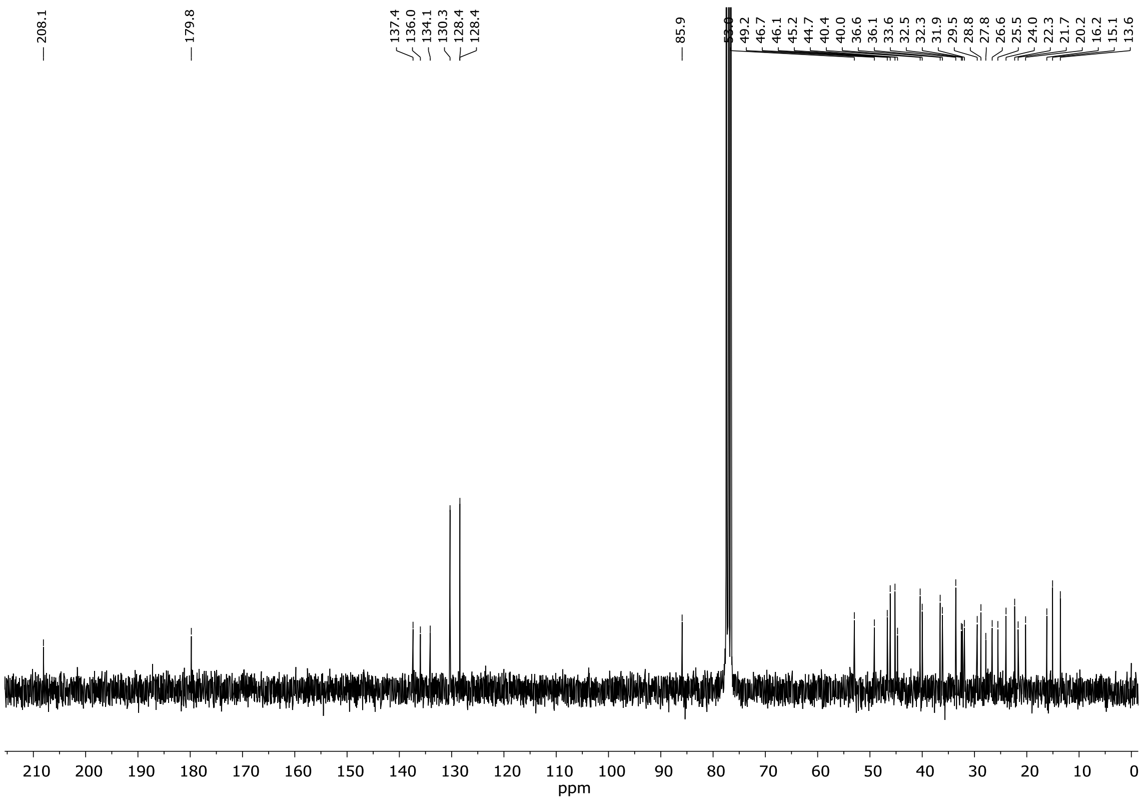

Figure S15. ^{13}C NMR spectrum of (*E*)-2-benzylidene-19,28-epoxyoleanane-3,28-dione (**4a**) (75.47 MHz, $CDCl_3$).

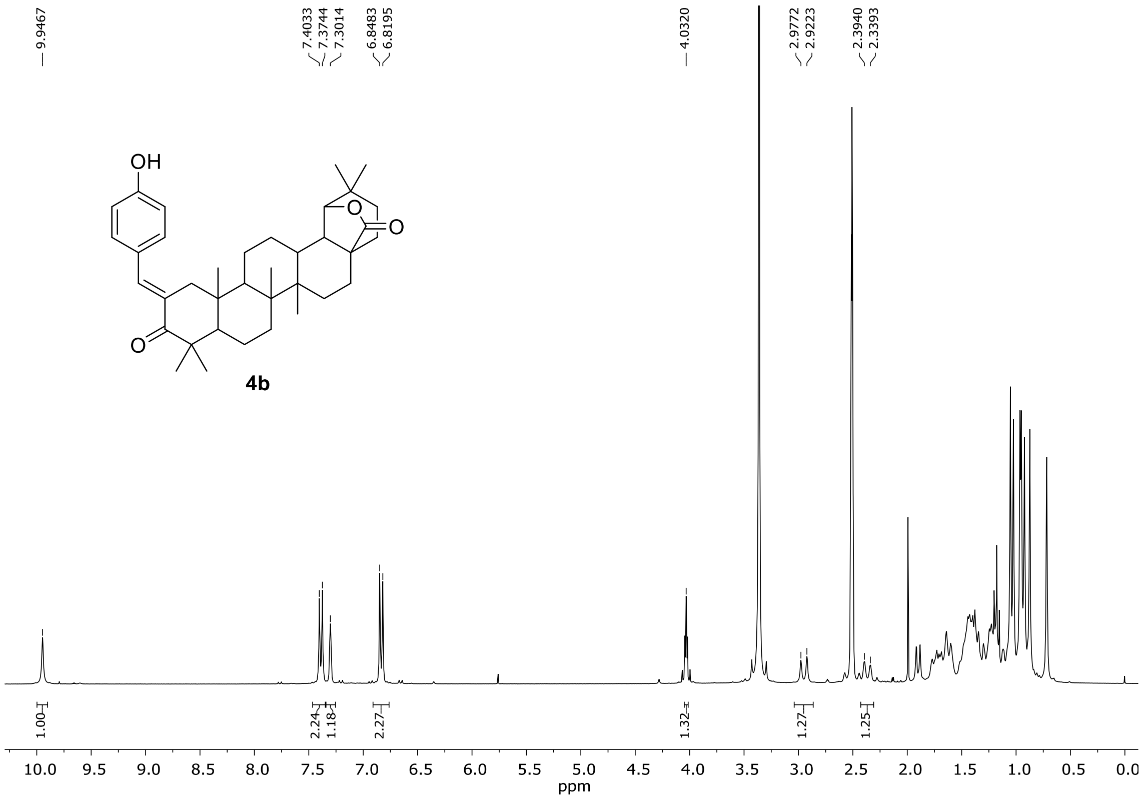

Figure S16. ^{1}H NMR spectrum of (*E*)-2-(4-hydroxybenzylidene)-19,28-epoxyoleanane-3,28-dione (**4b**) (300.13 MHz, DMSO-d_6).

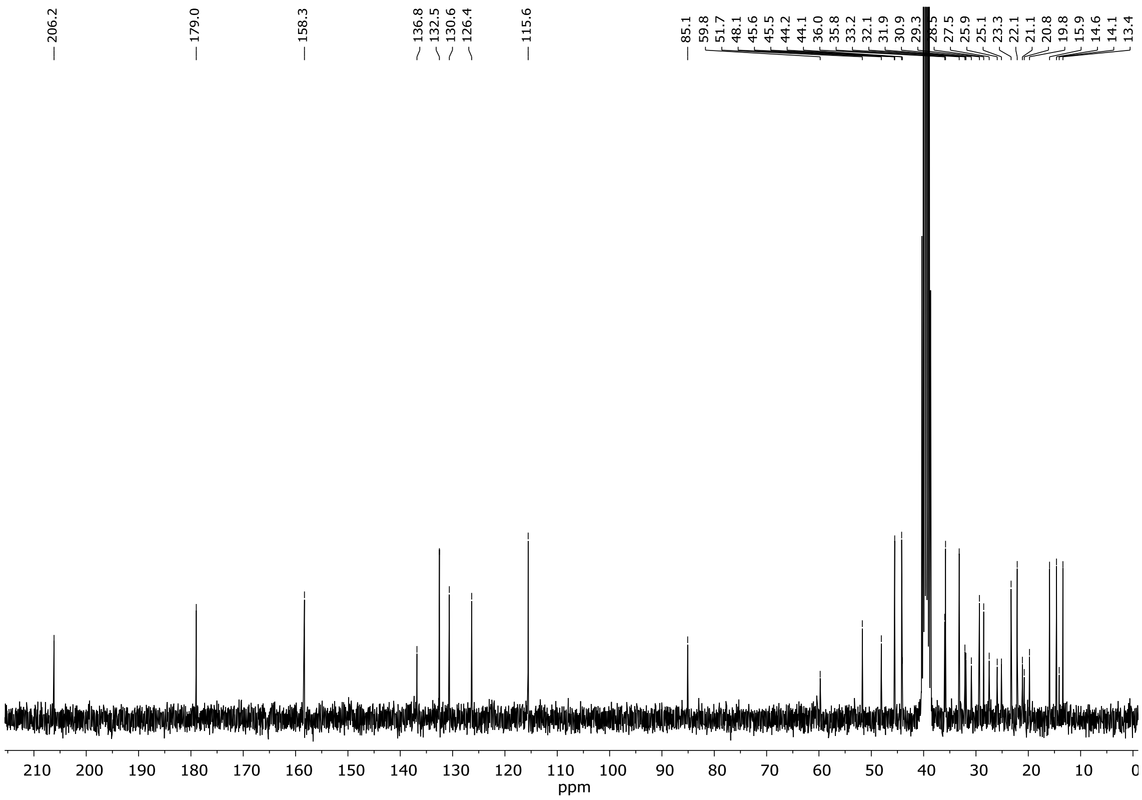

Figure S17. ^{13}C NMR spectrum of (*E*)-2-(4-hydroxybenzylidene)-19,28-epoxyoleanane-3,28-dione (**4b**) (75.47 MHz, DMSO-d_6).

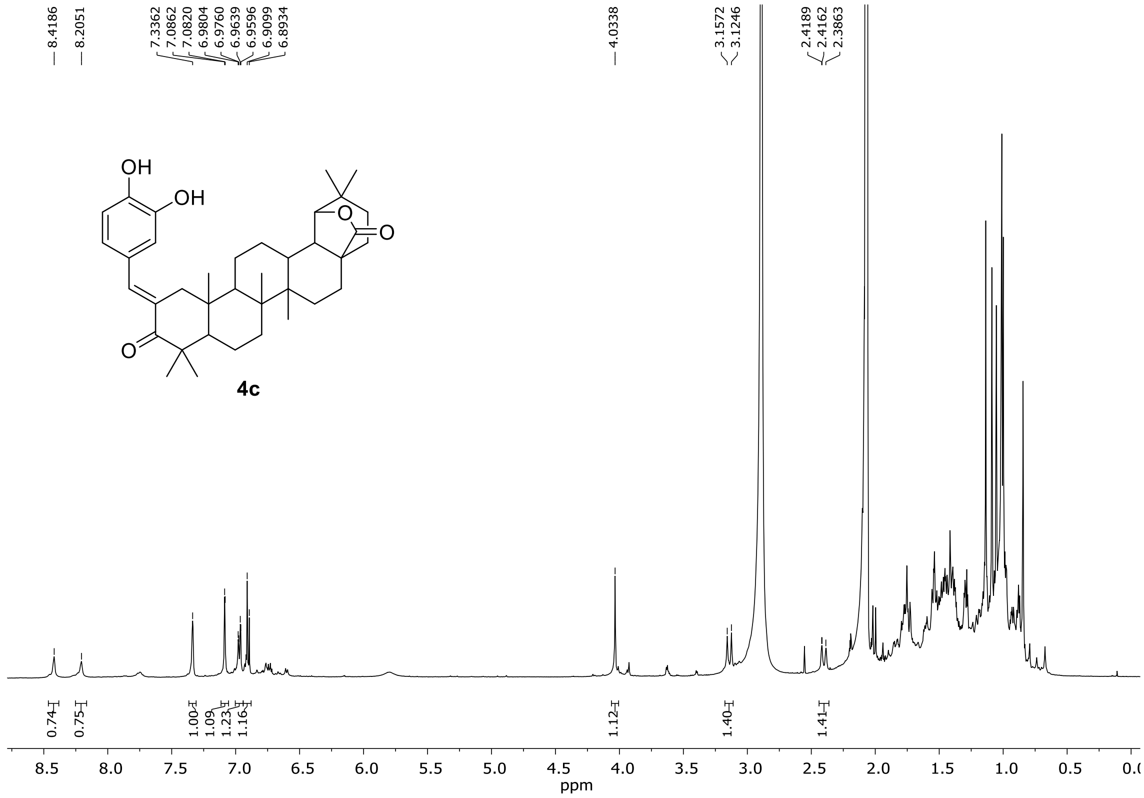

Figure S18. ^{1}H NMR spectrum of (*E*)-2-(3,4-dihydroxybenzylidene)-19,28-epoxyoleanane-3,28-dione (**4c**) (500.13 MHz, Acetone-d_6).

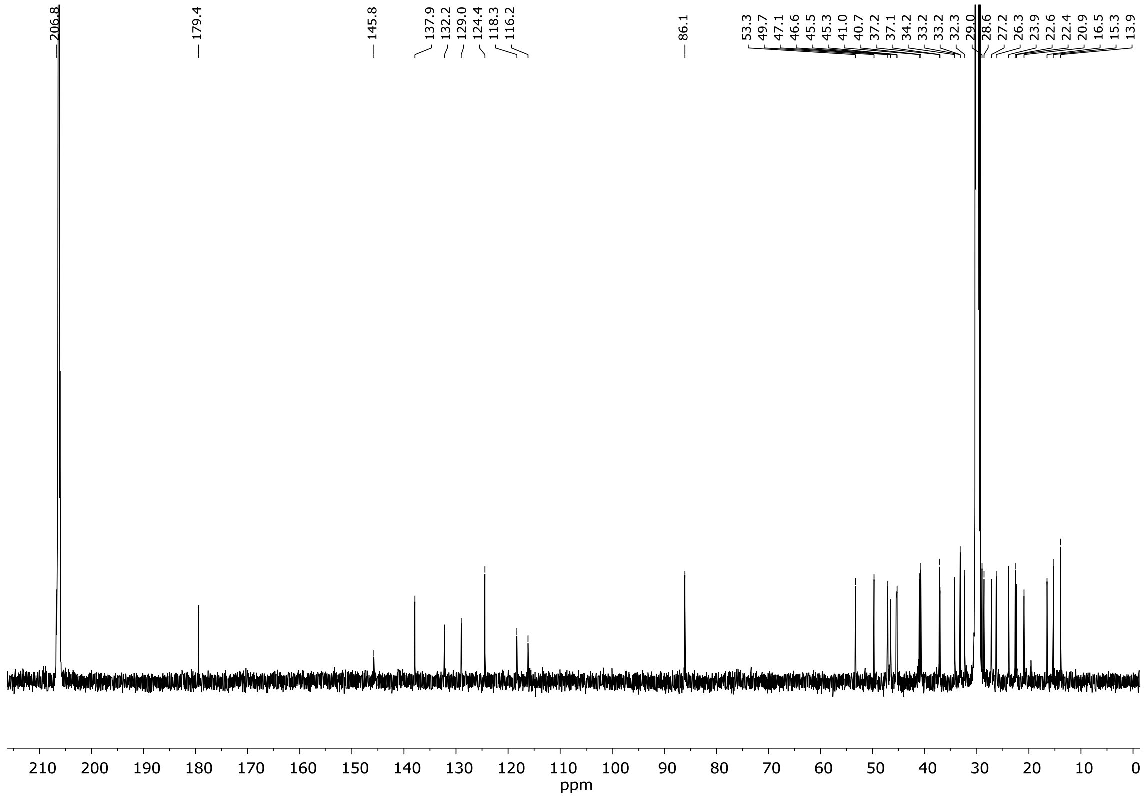

Figure S19. ^{13}C NMR spectrum of (*E*)-2-(3,4-dihydroxybenzylidene)-19,28-epoxyoleanane-3,28-dione (**4c**) (125.77 MHz, Acetone-d_6).

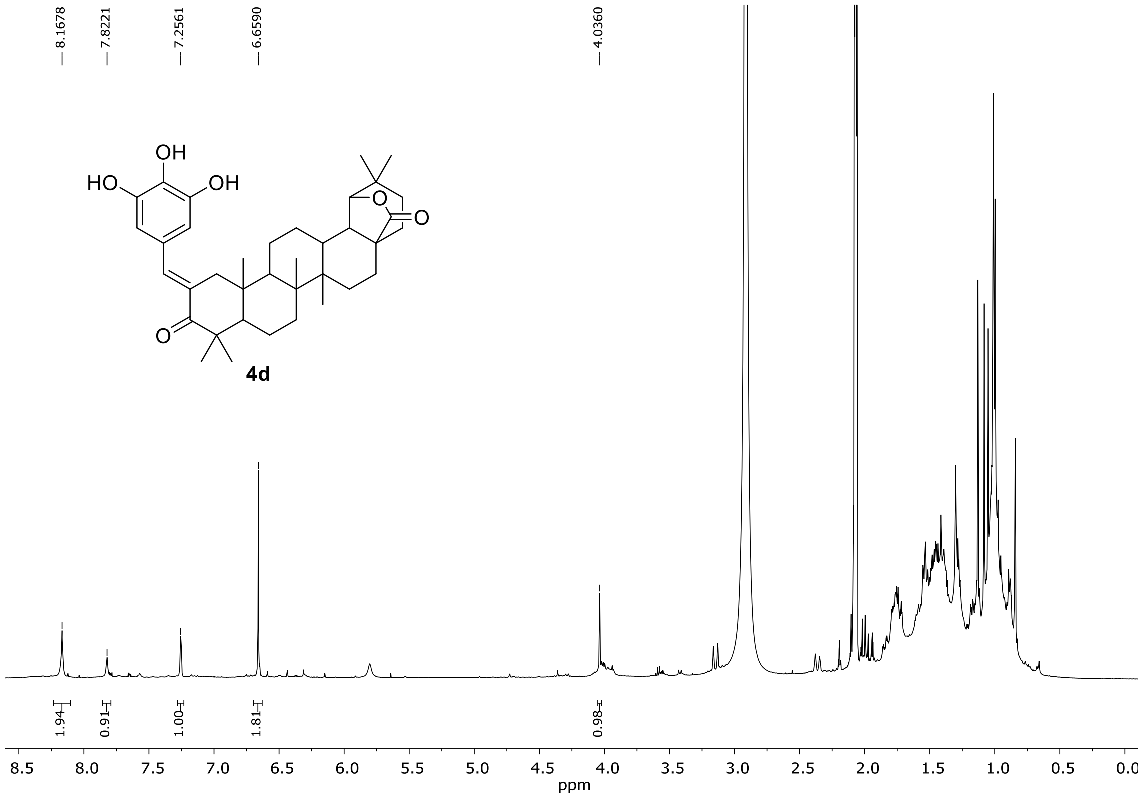

Figure S20. ^{1}H NMR spectrum of (*E*)-2-(3,4,5-trihydroxybenzylidene)-19,28-epoxyoleanane-3,28-dione (**4d**) (500.13 MHz, Acetone-d_6).

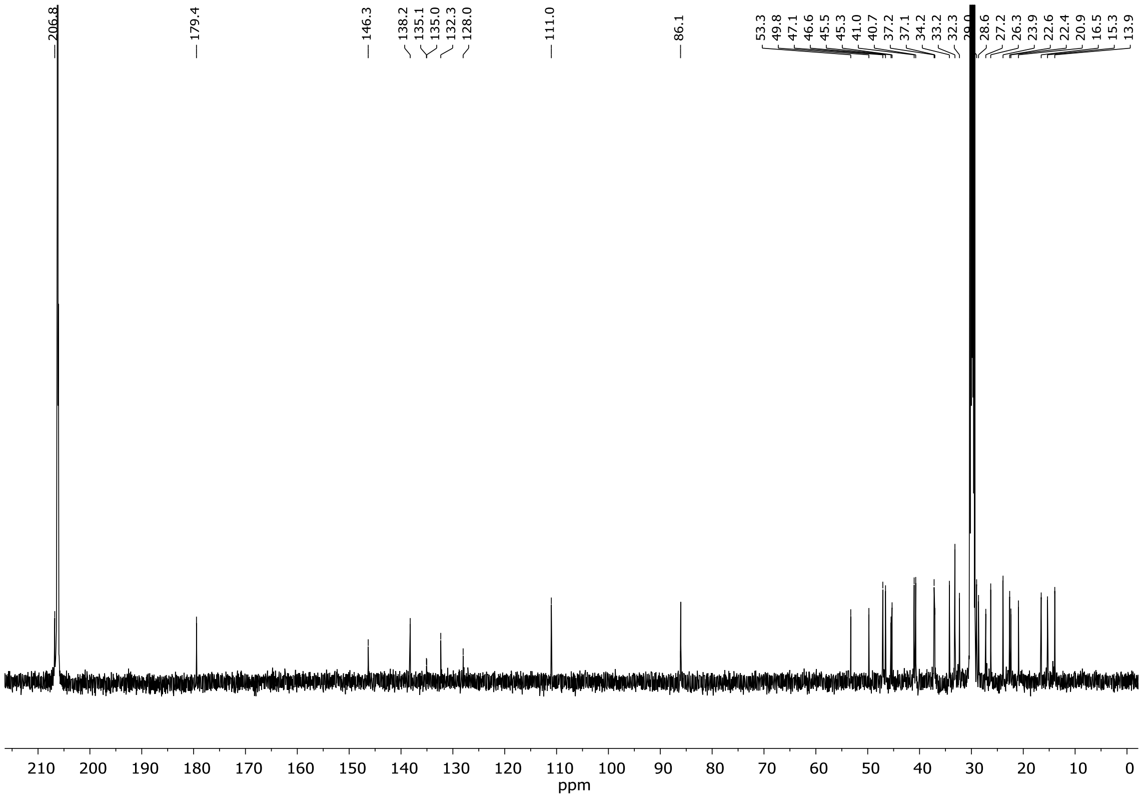

Figure S21. ^{13}C NMR spectrum of (*E*)-2-(3,4,5-trihydroxybenzylidene)-19,28-epoxyoleanane-3,28-dione (**4d**) (125.77 MHz, Acetone-d_6).

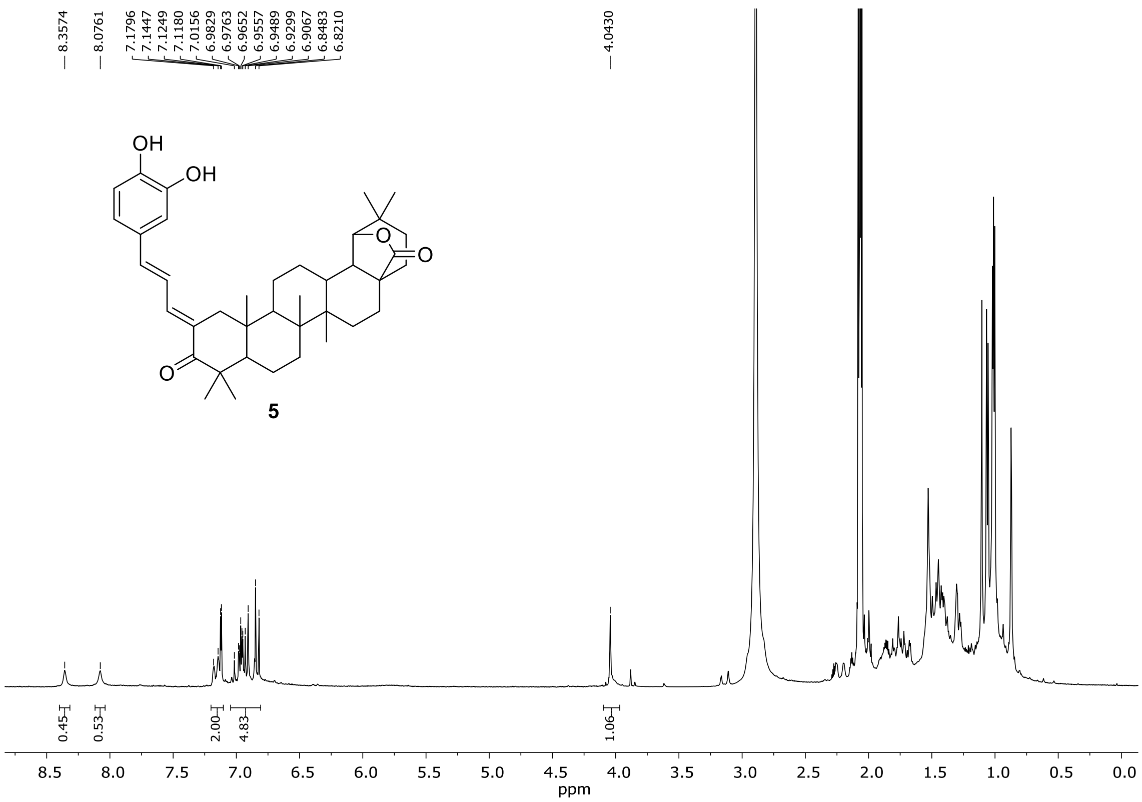

Figure S22. ^{1}H NMR spectrum of (*E*,*E*)-2-[3-(3,4-dihydroxyphenyl)allylidene]-19,28-epoxyoleanane-3,28-dione (**5**) (300.13 MHz, Acetone-d_6).

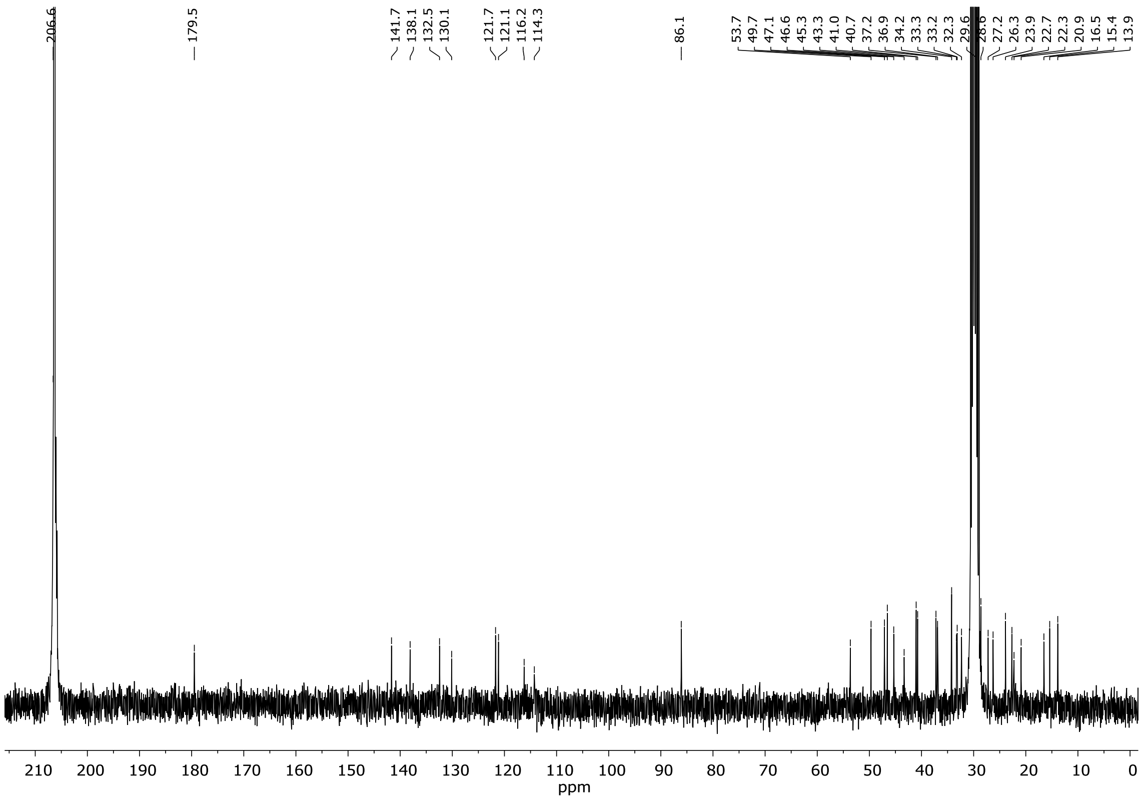

Figure S23. ^{13}C NMR spectrum of (*E*,*E*)-2-[3-(3,4-dihydroxyphenyl)allylidene]-19,28-epoxyoleanane-3,28-dione (**5**) (75.47 MHz, Acetone-d_6).

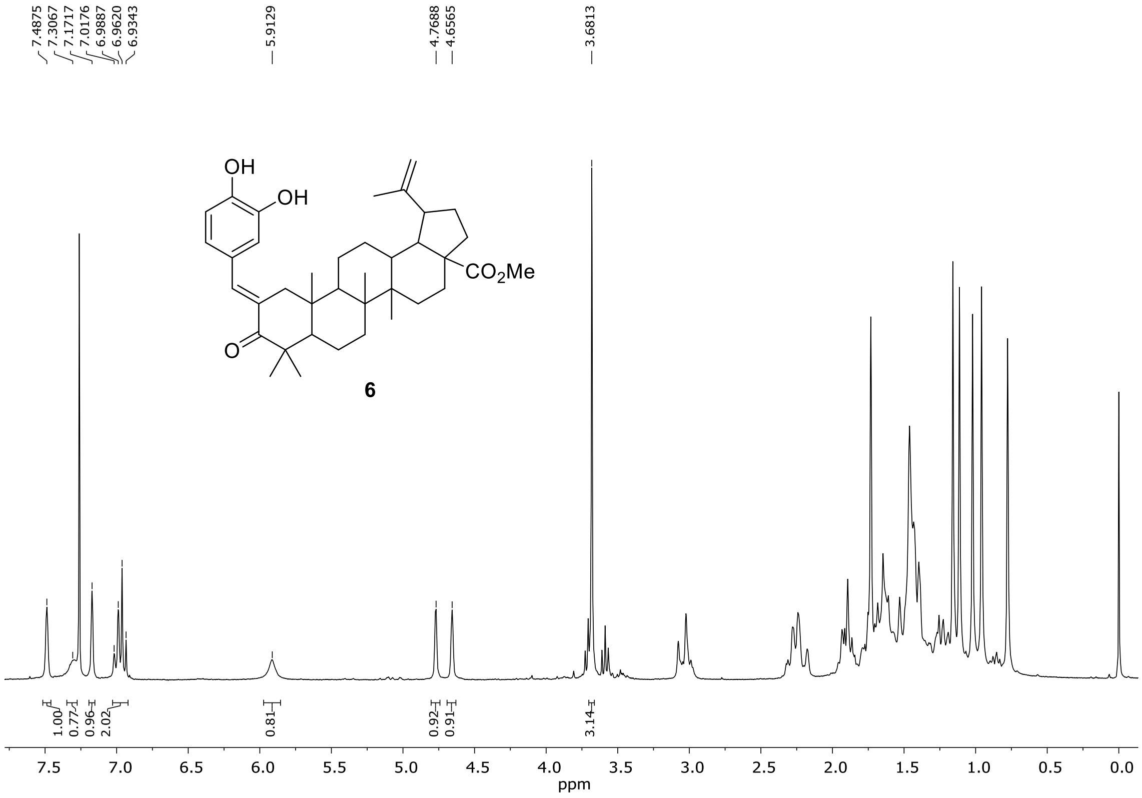

Figure S24. ^{1}H NMR spectrum of methyl (*E*)-2-(3,4-dihydroxybenzylidene)betulonate (**6**) (300.13 MHz, $CDCl_3$).

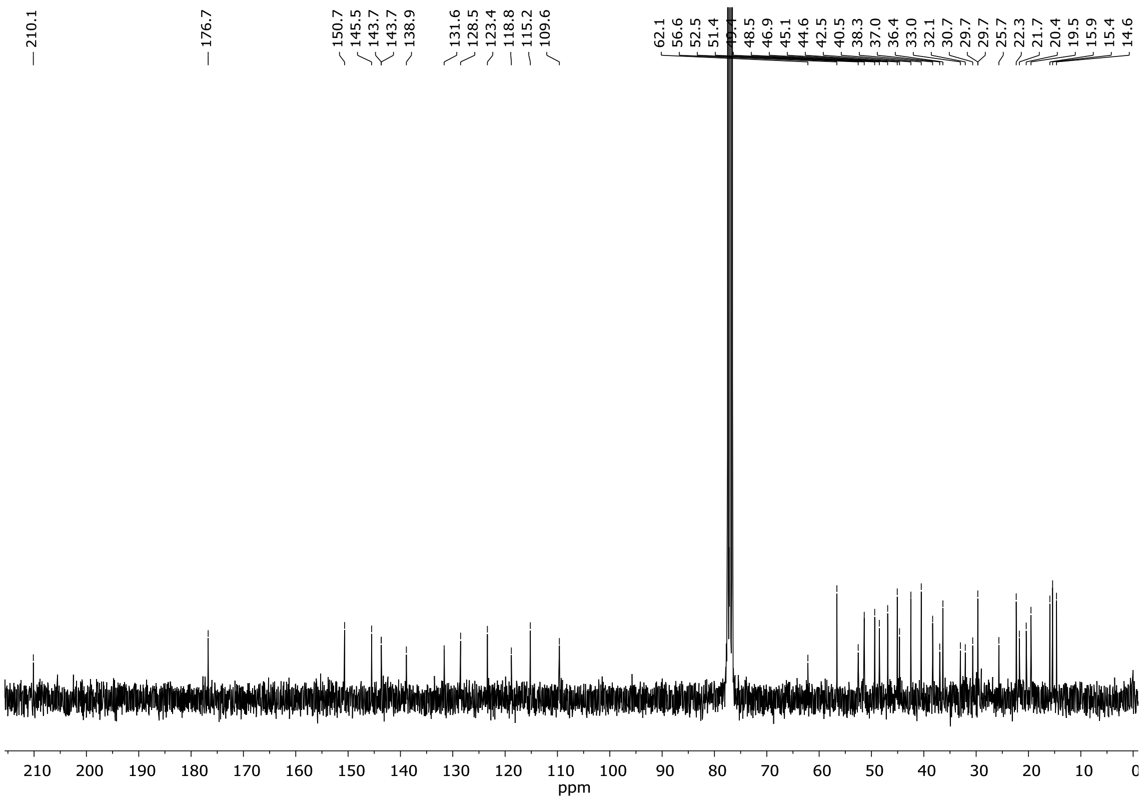

Figure S25. ^{13}C NMR spectrum of methyl (*E*)-2-(3,4-dihydroxybenzylidene)betulonate (**6**) (75.47 MHz, $CDCl_3$).
